# The Oct1 homolog Nubbin is a repressor of NF-κB-dependent immune gene expression that increases the tolerance to gut microbiota

**DOI:** 10.1186/1741-7007-11-99

**Published:** 2013-09-06

**Authors:** Widad Dantoft, Monica M Davis, Jessica M Lindvall, Xiongzhuo Tang, Hanna Uvell, Anna Junell, Anne Beskow, Ylva Engström

**Affiliations:** 1 Department of Molecular Biosciences, The Wenner-Gren Institute, Stockholm University, SE-106 91, Stockholm, Sweden; 2 Huddinge Genomics Core Facilities, Department of Biosciences and Nutrition, Karolinska Institute, SE-141 87, Huddinge, Sweden; 3 Present address: Laboratories for Chemical Biology Umeå (LCBU), Umeå University, SE-901 87, Umeå, Sweden; 4 Present address: Department of Pathology and Cell Biology, Columbia University, New York, USA

**Keywords:** Antimicrobial peptides, *Drosophila*, Gene regulation, Host-pathogen interaction, Immune signaling, Innate immunity, NF-kappaB, Oct /POU transcription factors, Stress response

## Abstract

**Background:**

Innate immune responses are evolutionarily conserved processes that provide crucial protection against invading organisms. Gene activation by potent NF-κB transcription factors is essential both in mammals and *Drosophila* during infection and stress challenges. If not strictly controlled, this potent defense system can activate autoimmune and inflammatory stress reactions, with deleterious consequences for the organism. Negative regulation to prevent gene activation in healthy organisms, in the presence of the commensal gut flora, is however not well understood.

**Results:**

We show that the *Drosophila* homolog of mammalian Oct1/POU2F1 transcription factor, called Nubbin (Nub), is a repressor of NF-κB/Relish-driven antimicrobial peptide gene expression in flies. In *nub*^*1*^ mutants, which lack Nub-PD protein, excessive expression of antimicrobial peptide genes occurs in the absence of infection, leading to a significant reduction of the numbers of cultivatable gut commensal bacteria. This aberrant immune gene expression was effectively blocked by expression of Nub from a transgene. We have identified an upstream regulatory region, containing a cluster of octamer sites, which is required for repression of antimicrobial peptide gene expression in healthy flies*.* Chromatin immunoprecipitation experiments demonstrated that Nub binds to octamer-containing promoter fragments of several immune genes. Gene expression profiling revealed that *Drosophila* Nub negatively regulates many genes that are involved in immune and stress responses, while it is a positive regulator of genes involved in differentiation and metabolism.

**Conclusions:**

This study demonstrates that a large number of genes that are activated by NF-κB/Relish in response to infection are normally repressed by the evolutionarily conserved Oct/POU transcription factor Nub. This prevents uncontrolled gene activation and supports the existence of a normal gut flora. We suggest that Nub protein plays an ancient role, shared with mammalian Oct/POU transcription factors, to moderate responses to immune challenge, thereby increasing the tolerance to biotic stress.

## Background

All multicellular organisms rely on evolutionarily selected immune defense mechanisms for protection against faster growing unicellular organisms, such as bacteria, fungi and protozoa, as well as viruses. In addition, multicellular parasites are threats that have to be combated by protective and reactive defense systems. The innate immune system, which is present in all metazoans, possesses the necessary duality in preventing infections and conquering them effectively. The former involves constitutively operating defenses, which are always present and counteract the invasion and growth of microbes, while the latter is based on recognition of the invader(s) and the immediate activation of cascades of parallel immune reactions with the purpose of eradicating the invading organism.

*Drosophila melanogaster* is a powerful model organism to identify genes involved in the innate immune system and its regulation. Several signal transduction pathways are involved in transferring information from the extracellular site of infection to elicit changes in gene expression of effector molecules such as antimicrobial peptides (AMPs) and reactive oxygen species (ROS) (reviewed in [1-5]). There are several families of AMP genes in *Drosophila* and other insects, most of which have been shown to be highly up-regulated in response to infection, primarily a result of signaling via the Toll and Immune Deficiency (IMD) pathways. These pathways are activated through extracellular recognition of pathogen-specific signature molecules, such as bacterial peptidoglycans. Signal transduction promotes nuclear translocation of the key NF-κB/Rel transcription factors *Dorsal*-related immunity factor (Dif) and Relish, which bind to a large number of target genes and activate their expression. As a result of transcription factor binding, dramatic up- and down-regulation of immune-regulated genes occurs, as was shown in several whole genome expression analyses (reviewed in [2,3]). Immune response genes contain infection-induced response elements (IRE), which typically consists of a few nested κB-like sites to which the NF-κB/Rel factors bind, linked with target sequences for tissue-specific GATA transcription factors [2]. Constitutive expression of ROS and of subsets of AMP genes is evident in epithelial linings of the digestive, respiratory and reproductive organs [4-6]. This is controlled via independent regulatory modules, of which a few have been identified and characterized [7,8].

Negative control of immune defense genes is of paramount importance to prevent aberrant activation, and to attenuate the immune response once the infection is eliminated. It has been shown that negative feedback regulation occurs at several levels of both the Toll and IMD pathways (reviewed in [3-5]). Blocking this negative feedback regulation leads to sustained and/or stronger immune reactions [3,9]. Direct transcriptional repression of immune defense genes has also been demonstrated. The homeodomain transcription factor Caudal (Cad) was shown to act as a gut-specific negative regulator of AMP gene expression [10], and *Drosophila* AP1 and STAT proteins were reported to act as negative regulators of *AttacinA* (*AttA*) expression [11]. However, continuous presence of commensal microbes in the gut does not promote constitutive activation of large batteries of genes in an NF-κB-dependent manner, therefore additional transcriptional regulators must exist that repress or modulate the expression of immune defense genes.

The POU family of transcription factors constitutes a large group arranged in six subclasses (I to VI) [12,13]. The name POU has its origin from the founding mammalian members Pit-1 and Oct-1/Oct-2, and the *Caenorhabditis elegans* Unc-86 protein [14]. Five different POU protein genes are present in the *Drosophila melanogaster* genome*,* belonging to four of the POU family subclasses, indicating that this transcription factor family is evolutionarily ancient [12,13]. The *Drosophila nub* gene (also called *POU domain protein 1* (*Pdm1*)) encodes a class II POU protein [15-17] and is a homolog of the human *POU2F1/Oct-1* and *POU2F2/Oct-2* genes [18], with which it shares considerable sequence similarity [15,17]. It has been predicted through genome annotation that the *nub* gene contains two independent transcription units (*nub-RB* and *nub-RD*) that each encode one specific protein variant: Nub-PB (104 kDa) and Nub-PD (65 kDa), which share the C-terminus, including the DNA-binding POU and homeodomains, but differ in their N-termini (Additional file 1). The *Drosophila nub* gene was originally identified as a viable mutation, *nub*^*1*^, caused by the insertion of a retrotransposon in the promoter region [19,20] just upstream of the first exon of the *nub-RD* transcription unit (Additional file 1). The *nub* gene has been extensively studied for its roles in embryonic development, in differentiation of the central nervous system [21-24], and for normal growth and patterning of wings and legs [20,25-28]. More recently, immunostaining of Nub protein has been used as an enterocyte cell marker in the adult midgut [29], but the role of *nub* in these cells has not been defined.

Mammalian POU factors are well known regulators of genes involved in both innate and adaptive immune processes. Initially, the mammalian class II factors Oct-1 and Oct-2 (Oct1/2) were identified as activators of immunoglobulin gene expression in B cells via octamer sequences [30]. Although the involvement of Oct-1/2 in immunoglobulin gene expression and B-cell development could not be confirmed in knock-out mice [31,32], a large number of immunomodulatory and inflammatory genes have been shown to be targets of Oct-1 *in vitro* and in cell-based assays (reviewed by [33,34]).

We previously isolated cDNAs for three POU domain transcription factors in a yeast screen for novel regulators of immune response genes [35]. One of these, corresponding to the *nub-RD* transcript, activated expression of a *Cecropin A1-luciferase* (*CecA1-luc*) reporter in *Drosophila* cells, indicating that the Nub-PD protein is able to bind and regulate transcription from the *CecA1* promoter. Here we show that Nub-PD acts primarily as a negative regulator of immune defense genes. This negative control of effector gene expression seems to be crucial in supporting a normal gut microbiome, as mis-regulated gene expression, due to a mutation in the *nub* gene, significantly changed the commensal gut flora. The elevated AMP gene expression was NF-κB/Relish-dependent, and could be blocked by transgene expression of Nub-PD, demonstrating its capacity to directly down-regulate expression of immune defense genes. We suggest that Nub protein serves a crucial role in suppressing aberrant immune and stress gene activation in healthy flies, thereby promoting immune homeostasis and tolerance to the commensal microflora.

## Results

### Nub is a negative regulator of NF-κB/Relish-driven immune gene expression

We isolated a *nub* cDNA in a yeast screen for regulators of the *Drosophila CecA1* gene [35]. This cDNA represented the *nub-RD* transcript, expressed from the *nub* gene, encoding the Nub-PD protein form (Additional file 1). In *Drosophila* cell culture transfection assays, the *nub-RD* cDNA was found to activate expression of a *CecA1-luc* reporter construct [35], supporting a possible role as a regulator of immune response genes*.* This result prompted an in-depth investigation of the *in vivo* role of this POU transcription factor in regulation of immune gene expression in flies.

**Figure 1:**
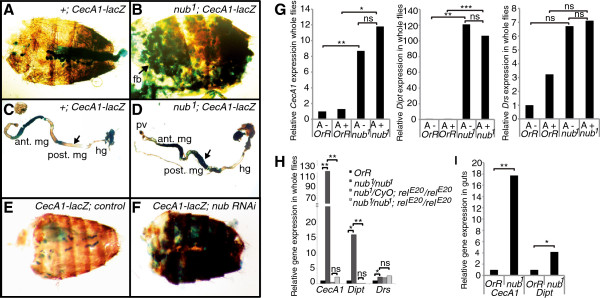
**Antimicrobial peptide genes are over-expressed in uninfected *nub***^***1 ***^**flies. (A**-**F)** Reporter β-galactosidase staining (*CecA1-lacZ*) in fat body and intestine of uninfected flies. Strong β-galactosidase expression was observed in fat body and posterior midgut (arrows) in *nub*^*1*^ mutant background **(B**,**D)** but not in wild type background **(A**,**C)**. Elevated reporter gene expression was confirmed using RNA interference against *nub* in the fat body (*CecA1-lacZ/c564-Gal4;UAS-dsnub/+*) **(F)**, in comparison with control flies (*CecA1-lacZ/c564-Gal4; +/+*) **(E)**. **(G**,**H)** Quantification of *CecA1*, *Dipt* and *Drs* mRNA levels in extracts of whole flies by RT-qPCR after pre-treatment with a cocktail of antibiotics (A+) or untreated control (A–) **(G)**, and in *Rel*^*E20*^ mutant background **(H)**. **(I)** Quantification of *CecA1* and *Dipt* mRNA levels by RT-qPCR in extracts of dissected intestines. The data are mean values; *n =* 3 **(G)**, *n =* 4 **(H**,**I)**. Statistical significance was calculated using paired *t*-test, *P <0.05, **P <0.01, ***P <0.001. fb, fat body; hg, hind gut; mg, midgut; pv, proventriculus; ns, not significant.

To examine if Nub-PD is necessary for *CecA1* expression *in vivo*, we analyzed the expression of a *CecA1-lacZ* reporter gene in uninfected flies, in a wild-type (wt) and *nub*^*1*^ mutant background*.* In homozygous *nub*^*1*^ flies (Additional file 1), expression of the *nub-RD* transcript is strongly reduced [20] and no Nub-PD protein is detected on immunoblots (Additional file 2B), whereas the Nub-PB protein is still expressed in the *nub*^*1*^ flies, confirming that *nub*^*1*^ mutation is specifically affecting the expression of the Nub-PD protein. Surprisingly, we observed prominent β-galactosidase (β-gal) reporter staining in the fat body of uninfected *nub*^*1*^*; CecA1-lacZ* flies (Figure 1B) compared to the control flies (Figure 1A), indicating that Nub-PD acts as a negative regulator of *CecA1* expression in fat body. In addition, we observed strong *CecA1-lacZ* expression in the posterior midgut of *nub*^*1*^*; CecA1-lacZ* flies (compare Figure 1C and D; arrows). To confirm that the elevated β-gal reporter staining is a consequence of the *nub*^*1*^ mutation, we used RNA interference (RNAi) to down-regulate *nub* transcript and protein. Expression of a hairpin construct, *UAS-dsnub*, driven by a fat body-specific Gal4 driver line (*c564-Gal4*) in transgenic flies, combined with the *CecA1-lacZ* reporter, promoted strong β-gal reporter staining in the fat body, compared to control flies (Figure 1E,F), strongly supporting that the *nub* gene is responsible for repression of *CecA1* in healthy flies*.*

These results were further substantiated by analyzing the steady-state mRNA levels of a few AMP genes in extracts of whole flies and dissected guts by quantitative reverse transcriptase-PCR (RT-qPCR) (Figure 1G-I). Expression of *CecA1* and *Diptericin* (*Dipt*) was significantly higher in *nub*^*1*^ than in wt flies (Figure 1G-1I), whereas *Drosomycin (Drs)* expression was only slightly increased (Figure 1G,H). To eliminate the possibility that the high level of AMP gene expression was due to the presence of an ongoing infection, and not the *nub*^*1*^ mutation, we treated flies with a potent cocktail of antibiotics prior to the analysis. Antibiotic-treated and untreated flies promoted similar levels of high *CecA1* and *Dipt* expression (Figure 1G), indicating that Nub-PD was responsible for the repression of the expression of at least these two AMP genes, and also suggesting that activation, that is*,* de-repression, occurs both in the absence and presence of commensal microbes in healthy flies. The *CecA1* and *Dipt* genes are well-established targets of NF-κB/Relish transcriptional activation. Importantly, we found that the high steady-state expression of *CecA1* and *Dipt* in uninfected *nub*^*1*^ flies is Relish*-*dependent, because it was significantly reduced in *Rel*^*E20*^ mutants (Figure 1H), whereas the small but significant up-regulation of *Drs* in *nub*^*1*^ flies was Relish-independent. Taken together, this indicates that Nub-PD acts as a transcriptional repressor of Relish-dependent genes, possibly rendering these genes inactive in the absence of infection. In accordance with this assumption, we found that in the midgut, where Relish was previously shown to be constitutively cleaved and activated [10], *CecA1-lacZ* reporter staining was prominent in *nub*^*1*^ mutants (Figure 1D). In addition, *CecA1* and *Dipt* mRNA levels were significantly elevated in dissected guts from *nub*^*1*^ flies compared to wt controls (Figure 1I). We conclude that Nub-PD acts as a negative regulator of Relish-dependent *CecA1* and *Dipt* gene expression both in the gut and fat body. In response to bacterial infection, expression of *CecA1*, *Dipt* and *Drs* mRNAs was potently activated both in wt control and *nub*^*1*^ flies (Additional file 3). The expression levels of all three genes were reproducibly higher in *nub*^*1*^ compared to wt, suggesting a negative effect of the presence of Nub-PD in wt flies. However, due to large biological variation in transcriptional response to infection, especially in the *nub*^*1*^ mutant, the difference was not statistically significant (Additional file 3). In summary, our results indicate that Nub-PD is a negative regulator of the expression of at least two AMP genes, *CecA1* and *Dipt*, in healthy flies. In response to infection, activation is dominant and repression is to a large extent alleviated, leading to strong immune gene expression in both wt and *nub*^*1*^ flies; at the same time, some de-repression was evident in *nub*^*1*^ mutants.

### The commensal bacterial load is significantly diminished in guts of *nub*^*1*^ mutant flies

**Figure 2:**
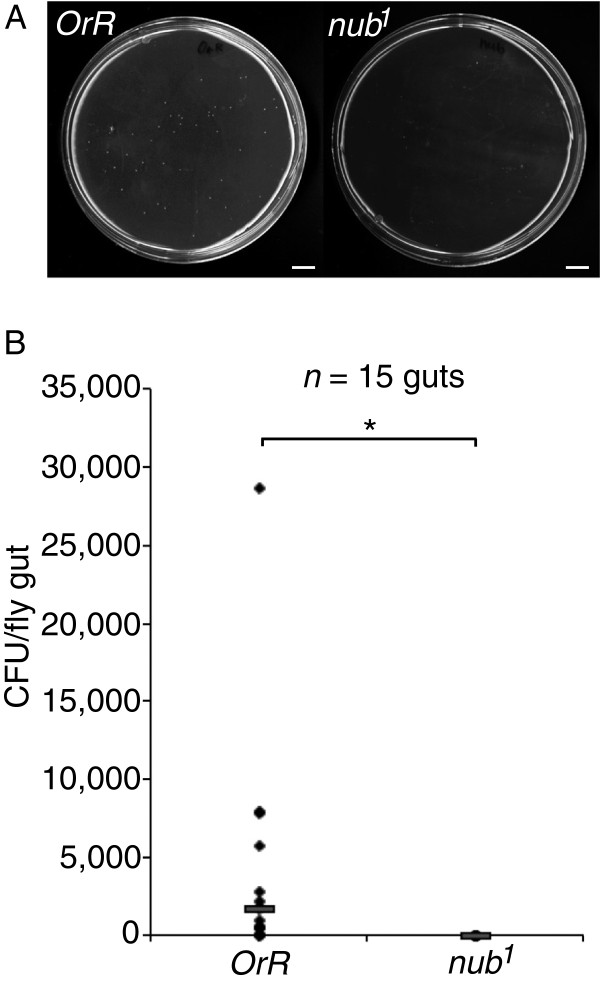
**Homogenates of *nub***^***1 ***^**mutants contain a reduced number of platable bacteria. (A)** Homogenates of dissected *nub*^*1*^ guts, spread out on standard lysogeny broth agar plates, contain a significantly reduced number of platable colony forming units compared to diluted homogenates from guts of *OrR* controls. **(B)** A total of 15 individuals guts per fly strain (n) were assayed and the median of the experiment is shown in the graph as a horizontal bar. Statistical significance was calculated using unpaired *t*-test, *P <0.05. Scale bar in A = 1 cm. CFU, colony forming units.

A possible consequence of the elevated AMP gene expression in guts of *nub*^*1*^ flies is that the numbers of commensal bacteria would be reduced. To test this we plated the gut content from individual flies on bacterial medium (Figure 2A) and found that whereas wt flies typically contained between 10^3^ to 10^4^ colony forming units per gut, no bacterial colonies grew from the guts of *nub*^*1*^ mutants (*n =* 15) (Figure 2), indicating that *nub*^*1*^ guts constitute a highly bacteriostatic environment. Although no colonies grew, we do not consider the guts of *nub*^*1*^ mutants to be completely devoid of bacteria, because amplification of bacterial 16S rRNA genes indicated that guts from *nub*^*1*^ mutants do contain bacteria (unpublished). Our conclusion is, however, that the numbers of live, cultivatable bacteria in guts of *nub*^*1*^ mutants is strikingly diminished compared with that of wt flies. Thus, in the absence of a functional Nub-PD protein as in *nub*^*1*^ mutants, the gut microbiome is severely affected, most likely as a consequence of the increased expression of AMPs and possibly of other immune effector molecules.

### Nub-PD expression in *nub*^*1*^ flies is sufficient to restore a repressed status of target gene expression

**Figure 3:**
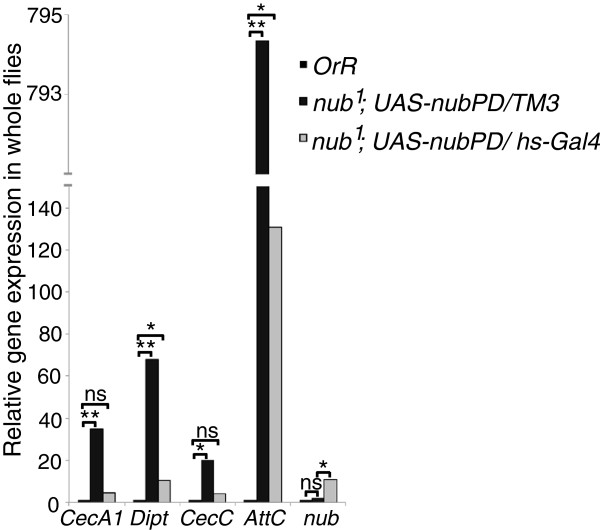
**Expression of Nub-PD from a transgene restores normal regulation of antimicrobial peptide genes in a *nub***^***1 ***^**mutant background.** Quantification of *CecA1*, *Dipt*, *CecC*, *AttC* and *nub-RD* mRNA by RT-qPCR in extracts of uninfected whole flies. Expression was measured in wild type (*OrR*; black bars), *nub*^*1*^ flies (*nub*^*1*^*/nub*^*1*^*; UAS-nub-RD/+*; dark gray bars) and *nub*^*1*^ flies expressing a *nub-RD* transgene (*nub*^*1*^*/nub*^*1*^*;UAS-nub-RD/hs-Gal4;* light gray bars). The data are mean values; *n =* 3*.* Statistical significance was calculated using paired *t*-test, *P <0.05, **P <0.01.

To investigate directly if Nub-PD acts as a transcriptional repressor of immune system genes, we over-expressed Nub-PD from a *UAS-nub-RD* construct in *nub*^*1*^ mutant background. We first analyzed expression of four AMP genes, *CecA1*, *CecC*, *Dipt* and *AttC*, in *nub*^*1*^*; UAS-nub* control flies, confirming strong over-expression of all four genes (Figure 3). A number of different *Gal4* driver lines were then tested: the use of strong/ubiquitous drivers was lethal whereas tissue-specific drivers only promoted marginal *nub-RD* over-expression. Using a heat-shock promoter-driven *Gal4* (*UAS-nub-RD/hs-Gal4*) it was also not possible to achieve high levels of *nub* mRNA expression after heat-shock induction, although several different experimental regimes were tested. It is unclear why this is the case, but it may indicate that strong over-expression of *nub-RD* results in negative feedback regulation, most likely at the post-transcriptional level. However, approximately 10-fold over-expression of *nub-RD* was reached routinely using leaky expression from the same transgene combination (*UAS-nub-RD/hs-Gal4*) (Figure 3). This relatively moderate level of *nub-RD* over-expression in a *nub*^*1*^ mutant background (*nub*^*1*^*; UAS-nub-RD/hs-Gal4*) was sufficient to reduce *CecA1*, *Dipt*, *CecC* and *AttC* expression levels by 50% to 80% compared to *nub*^*1*^*; UAS-nub-RD/TM3* control flies (Figure 3), demonstrating that Nub-PD acts directly as a negative regulator of gene expression. It also confirms that aberrant expression of these immune genes in *nub*^*1*^ flies is truly due to the lack of a functional Nub-PD protein and not caused by the genetic background of the stock, as expression of *UAS-nub-RD* was sufficient to restore the repressed status of these genes. In infected flies, over-expression of *nub-RD* did not reveal a significant effect on the expression of *CecA1* and *Dipt* (data not shown). As suggested above, this indicates that Nub-PD repression is relieved during infection.

### Repression of *CecA1* by Nub-PD requires an upstream Oct motif cluster

**Figure 4:**
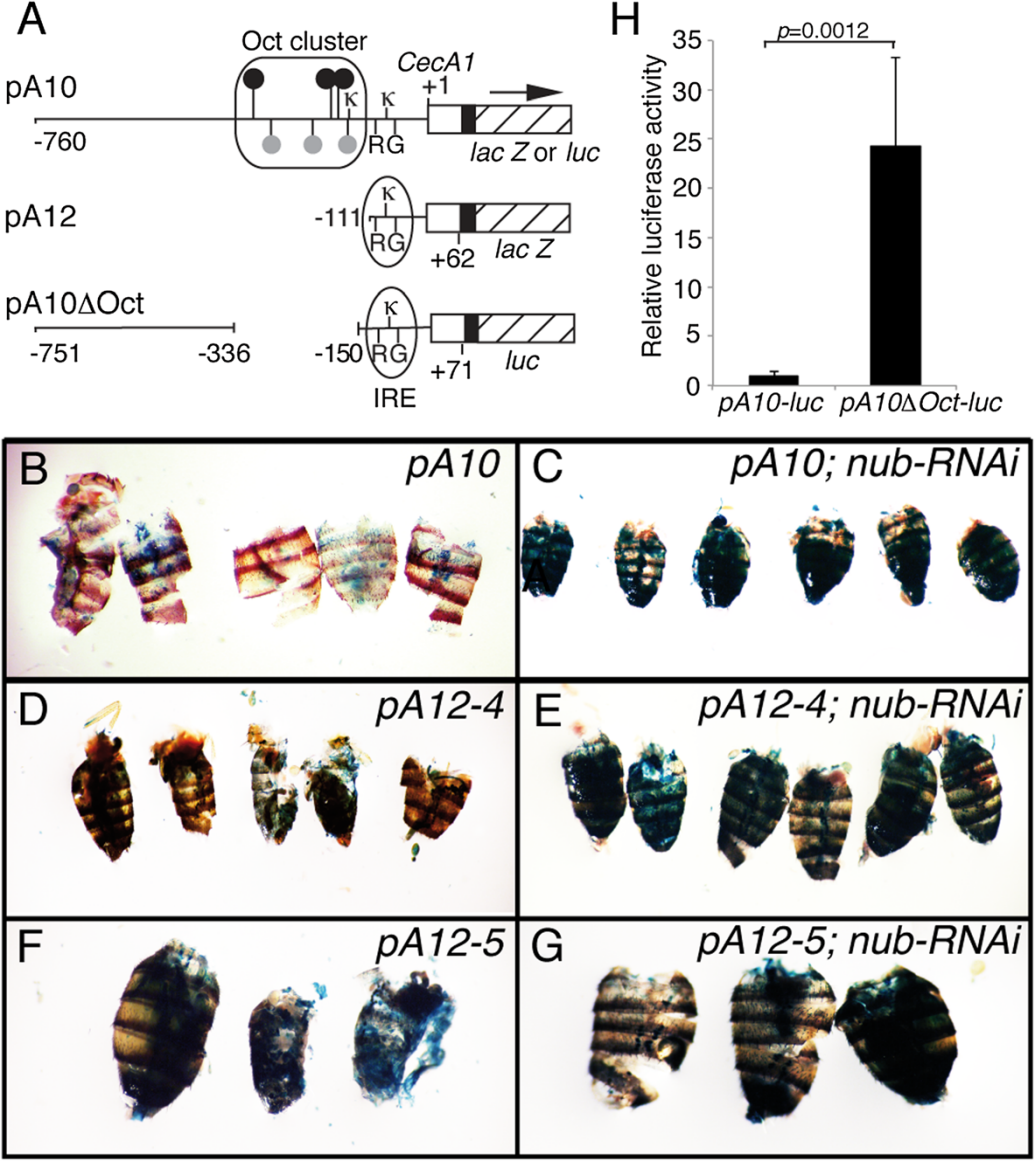
**A distal promoter region of *CecA1 *is required for Nub-PD-dependent repression. (A)** Schematic representation of the *CecA1-lacZ* and *CecA1-luc* constructs, either carried by transgenic *Drosophila* or used for cell transfections. The pA10 construct contains 760 or 751 bp of 5′ upstream region from the *CecA1* gene (horizontal line), and 62 or 71 bp of 5′ UTR (open box) fused to a SV40 leader (filled box), providing a translational start site in frame with the *Escherichia coli lacZ* or *luc* coding sequence (hatched box) [7,41]. Numbers refer to positions relative to the transcription start site (+1). Location of regulatory sequence motifs, as indicated by symbols and letters, is in scale. A previously characterized infection-induced response element (IRE) contains a κB-like site (κ), GATA site (G) and R1 site (R), and one additional κB site is located just 5′ of the IRE. A cluster of Oct sequence motifs (rectangle) contains several consensus Oct sequences (black circles) and Oct-like (gray circles) sequences (see Additional file 11 for sequences and exact locations). The pA12 construct contains 111 bp of 5′ sequence including the IRE, but not the Oct cluster. The pA10∆Oct-luc construct has an internal deletion of the whole Oct cluster (−336 to −150) but is otherwise identical to the pA10-luc construct. **(B**-**G)***CecA1-*driven β-gal staining in the fat body of abdomens from flies carrying either the pA10 construct (*pA10 CecA1-lacZ / TM3*) **(B**,-**C)** or the pA12 construct (*pA12 CecA1-lacZ*) **(D**-**G)**. Two independent transgenic lines, pA12-4 **(D**,**E)** and pA12-5 **(F**,**G)** were used. **(H)** Transfection of mbn-2 cells with *CecA1-luc* constructs confirms that the Oct cluster region is involved in repression of the *CecA1* promoter. The graph shows the mean values of relative luciferase activity and standard deviation (*n =* 6). Statistical significance was calculated using paired *t*-test, *P* = 0.0012.

We have previously reported that the upstream regulatory region of the *CecA1* gene contains both positively and negatively acting regulatory elements, based on experimental evidence from promoter-reporter constructs in transgenic flies [36]. Similar to mammalian Oct1/2 transcription factors, Nub-PD has been shown to bind with high affinity to the Oct consensus motif (ATGC^A^/_T_AAT) and to several closely related motifs [37-39] as well as more divergent non-Oct motifs [40]. We searched the *CecA1* upstream region for these sequence motifs and found that it harbors an Oct cluster, containing three Oct consensus motifs, two Oct-like/nub motifs of the type present in the *Drosophila choline acetyltransferase* gene [38] and one variant of the latter overlapping with a κB site, located just 5′ of the IRE (Figure 4A). We did not find any sequences matching the Nub binding sites (non-Oct type) reported to be responsible for repression of the *vestigial* gene [40].

To investigate if the upstream region containing the Oct cluster is involved in repressing *CecA1* expression in uninfected flies, we analyzed the expression of *CecA1-lacZ* constructs in transgenic flies carrying either a complete upstream region (pA10) or with a 5′ deletion, lacking the whole Oct cluster (pA12) (Figure 4A). Both constructs contain the IRE, which has previously been shown to contain necessary target sequences for NF-κB and GATA transcription factors [2], and to promote strong reporter gene expression in response to infection [36]. As a positive control, we analyzed the expression of *CecA1-lacZ* in flies in which *nub* had been down-regulated by RNAi in the fat body (*c564-Gal4; UAS-dsnub*)*.* This promoted strong reporter gene expression in the fat body of flies exposed to RNAi (Figure 4C) compared with the matched control flies, in which RNAi was not induced (Figure 4B). Two independent transgenic lines carrying the pA12 *CecA1-lacZ* transgene conferred strong reporter gene expression in abdominal fat body (Figure 4D,F), demonstrating that in the absence of the upstream region including the Oct cluster, the *CecA1* promoter is constitutively active in the fat body. The staining was remarkably strong compared with the control pA10 *CecA1-lacZ* flies (Figure 4B), and similar to the pattern and reporter strength of pA10 *CecA1-lacZ* in *nub* RNAi flies (Figure 4C). It is important to note that the whole experiment was carried out with uninfected flies, confirming that the *CecA1* promoter normally is repressed in healthy flies but aberrantly activated in *nub*-depleted flies. Expression of pA12 *CecA1-LacZ* was equally strong in abdominal fat body regardless of whether the flies had been exposed to *nub*-RNAi (Figure 4E,G) or not (Figure 4D,F). This indicates that Nub-PD primarily represses *CecA1* expression via sequences present in the −760 to −111 bp region.

Next we created a *luc* reporter construct with an internal deletion of the Oct cluster region from −336 to −150 (*pA10∆Oct-luc*) (Figure 4A), and analyzed its expression in transiently transfected *Drosophila* mbn-2 cells. Specific deletion of the Oct cluster promoted 25-fold higher expression levels compared to the *pA10-luc* control (Figure 4H), clearly demonstrating that the Oct cluster region acts as a negative *cis-*regulatory element.

In conclusion, deletion of the Oct cluster strongly enhances expression from the *CecA1* promoter both in cell transfections and *in vivo.* In addition, deletion of the *CecA1* upstream region that contains the Oct cluster leads to excessive *CecA1-lacZ* expression in a very similar manner as down-regulation of *nub* by RNAi, suggesting that Nub-PD regulates *CecA1* by binding to the Oct cluster.

### Nub protein is expressed in fat body and midgut of uninfected flies

**Figure 5:**
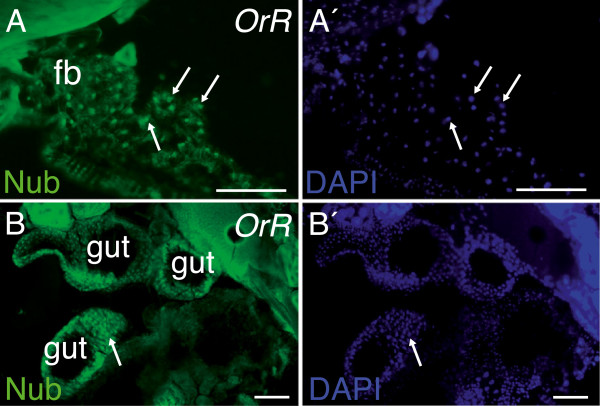
**Localization of Nub protein in immunocompetent tissues of flies. (A**,**B)** Immunostaining of Nub in frozen cryostat sections of uninfected *OrR* flies using a peptide-specific antibody directed against a common epitope of Nub-PB and Nub-PD protein. Nuclei stained with 4′-6-diamidino-2-phenylindole (DAPI) (A′-B′). Cryostat sections (20 μm thick) of adults showing nuclear (arrows) and cytoplasmic and staining of Nub in fat body tissue (A,A′) and in cross-sections of midgut (B,B′). Scale bars, 100 μm.

It has previously been shown that *nub* is expressed in the developing central nervous system of embryos [23] and in wing, haltere and leg discs of third instar larvae [20]. Except for distinct localization in midgut enterocytes [29], it is not known if Nub protein is expressed in immunoresponsive tissues. We produced an affinity-purified peptide-specific antibody against Nub. Immunoblot experiments confirmed the specificity of the antibody and also validated the recent annotation of the *nub* gene to encode two proteins of 104 kDa (Pdm-PB) and 65 kDa (Nub-PD) (Additional files 1 and 2A). Strong immunostaining in wing and leg imaginal discs of third instar larvae confirmed localization of Nub protein in these tissues (Additional file 2C and unpublished). Immunostaining of adult tissues in cryosections of whole flies revealed that Nub protein is present in fat body (Figure 5A), midgut (Figure 5B) and testis (unpublished). This correlates well with reported expression of *nub* transcripts in adults [42]. Nub immunostaining was not restricted in its subcellular localization to either nucleus or cytoplasm, instead its nuclear-cytoplasmic localization varied in different regions of these tissues, possibly reflecting alternative states of transcriptional regulation. In conclusion, *nub* is expressed in immune-responsive tissues, such as fat body and midgut of healthy flies, which would enable Nub protein to act as a transcriptional repressor of immune genes in the absence of infection.

### Chromatin immunoprecipitation experiments show that Nub binds to antimicrobial peptide genes *in vivo*

**Figure 6:**
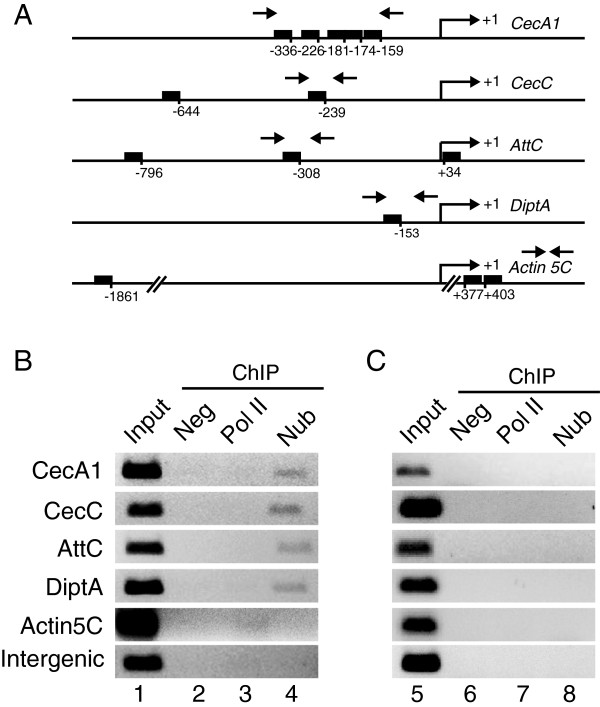
**Nub binds to the upstream region of several antimicrobial peptide genes. (A)** Promoter regions of *CecA1*, *CecC*, *Dipt*, *AttC* and *Act5C*, in which Oct and Oct-like-binding site(s) are represented by black boxes. The numbers below each box represents the position of the binding site in correlation to the transcriptional start site. The arrows indicate the forward and reverse primers. In the *Act5C* gene, two Oct-binding sites are located in the first intron, while the primers bind to a region in the second intron. **(B)** Chromatin was extracted from whole *OrR* flies after fixation with formaldehyde and immunoprecipitation reactions were carried out with a peptide-specific Nub antibody (lane 4). Negative control immunoprecipitations were carried out in parallel, either without antibody (lane 2) or with an antibody against the C-terminal domain of RNA polymerase II (lane 3), which is not expected to bind to the upstream region of these genes in uninfected flies. Primers located in the transcribed region of *Act5C* gene and in a non-transcribed region (intergenic region) were analyzed in parallel to assess the specificity of the chromatin immunoprecipitation, showing that RNA polymerase II, but not Nub, binds to the transcribed region of the *Act5C* gene, while neither protein binds to the non-transcribed region. **(C)** Control immunoprecipitation with chromatin, which was phenol-chloroform-extracted prior to fixation, showing the specificity of the protein-antibody interactions. As expected, no PCR products were observed with any of the antibodies.

*In vitro* DNA-protein interaction assays have previously demonstrated that Nub-PD binds with high affinity to the Oct sequence motif [37,39]. To analyze if Nub protein directly binds to the promoter regions of AMP genes, we carried out chromatin immunoprecipitation assays (ChIP)*.* The PCR primers used for amplification were located to cover one or several Oct sites in the 5′ regions of analyzed genes (Figure 6A). Nub protein bound to the promoter regions of *CecA1*, *CecC*, *AttC* and *DiptA* were isolated using the Nub-specific antibody (Figure 6B, lane 4). Importantly, Nub protein did not bind to the *Act5C* or to a non-transcribed intergenic region (Figure 6B, lane 4). Omitting the Nub antibody abolished the PCR product of all genes (Figure 6B, lane 2). As an additional control of specificity, we used an antibody against the C-terminal domain of RNA polymerase II, which is anticipated to neither bind to the relatively far 5′ upstream regions analyzed here nor to AMP gene promoters in uninfected conditions [43]. As expected, the C-terminal domain of RNA polymerase II antibody did not bind to any of the AMP gene promoters but showed weak binding to the *Act5C* transcription unit (Figure 6B, lane 3). Phenol-chloroform extraction of isolated chromatin prior to immunoprecipitation abolished the PCR product in all samples (Figure 6C, lanes 7 and 8), validating that the immunoprecipitation requires the presence of protein bound to DNA. This ChIP demonstrates that Nub protein physically interacts with the promoter region of several AMP genes, strongly indicating that this physical interaction is responsible for repression of these genes in the absence of infection.

### A large number of genes involved in immune system processes are up-regulated in *nub*^*1*^ flies

**Figure 7:**
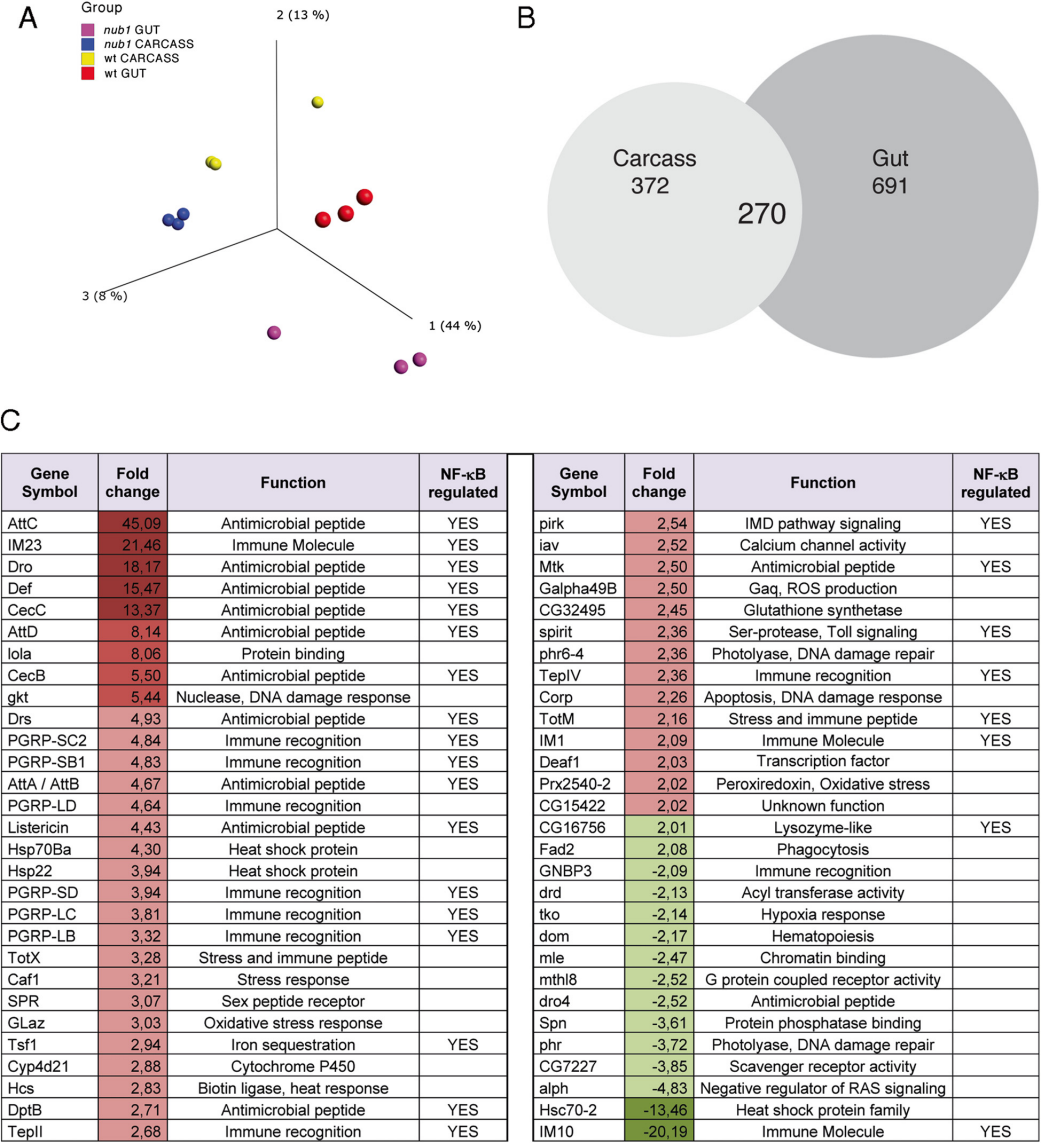
**Analysis of global mRNA expression profile in *nub***^***1 ***^**mutants compared to wild type flies. (A)** A factorial map of principal component analysis was conducted using all transcripts found to be expressed over background signal in at least one sample group. Samples are colored group-wise. The percentage found for each component in the plot can be interpreted as the variation seen within and between the different sample groups. For the principal component analysis plots, the largest explainable variance is found in component 1, where 44% of the data’s variation can be explained. This 44% is the biological difference found between the different tissues, gut or carcass. Component 2 with its 13% divides the data based on the biological variance explained by strain, that is, *nub*^*1*^ or wild type (wt). Component 3, with 8%, biologically nuances the data even further. **(B)** Venn diagram showing the overlap of carcass and gut differentially expressed genes in *nub*^*1*^ compared to wild type after removing background and filtering for fold change >2. **(C)** List of ‘Immune and Stress Response’ genes up- or down-regulated in *nub*^*1*^ mutants. Names and functions of the 58 genes constituting the enriched Biological Process cluster ‘Immune system and Response processes’ identified by a gene set enrichment analysis (Additional file 4) in carcass (without head and gut) from *nub*^*1*^ mutants. Fold change (mean values) of mRNA levels is indicated by numbers and color-coded (red, up-regulation; green, down-regulation). Genes that previously have been shown to be targets of the NF-κB/Relish/IMD pathway or a combination of NF-κB/Relish/IMD and NF-κB/Dif/Toll pathways [44-46] are indicated by ‘YES’.

To investigate if Nub-PD acts as a general repressor of immune gene transcription in healthy flies, we analyzed the global mRNA expression profile in *nub*^*1*^ mutants compared to wt flies by microarray analysis using Affymetrix Gene Chip *Drosophila* Genome 2.0 oligonucleotide arrays. Prior to mRNA extraction, the flies were dissected so that the two major immunoresponsive tissues, fat body (in carcass) and the digestive system (gut) were analyzed separately. The raw data were normalized, pre-processed and filtered to remove genes that were not expressed at a detectable level (see Methods). A factorial map of principal component analysis reveales that the individual biological samples cluster together within their own sample group, showing that the replicates are well represented as a population (Figure 7A). The variance seen within each sample group is biologically expected as each sample is a pooled set of flies. Sample groups are firstly separated on the basis of tissue (gut or carcass) and secondly on strain (*nub*^*1*^ or wt). No outlier samples were detected, hence all samples were included in further analysis. By comparing *nub*^*1*^ to the wt counterpart, either carcass or gut, we found 642 (carcass) and 961 (gut) transcripts to be differentially expressed by a fold change of two or higher. Out of these, 270 were differentially expressed in both carcass and gut (Figure 7B).

To further analyze the differentially expressed transcripts found in each sample type in *nub*^*1*^ versus wt, a gene set enrichment analysis was conducted identifying overrepresented Gene Ontology (GO) categories. The differentially expressed transcripts in carcass *nub*^*1*^ compared to wt are dominated by genes preferentially expressed in ‘Immune and Response processes’ (enriched with *P* <0.0002) (Additional files 4 and 5). Within this cluster of 58 genes (Figure 7C and Additional file 4) we find many well-characterized immune effector genes, such as AMPs and those involved in ROS production; immune recognition proteins, such as peptidoglycan recognition proteins (PGRPs); thioester proteins; and scavenger receptors. We also find genes involved in responses to abiotic stress, such as heat-shock proteins, detoxifying proteins, DNA damage repair, oxidative stress and wound healing. When we included *CecA1* and *Dipt*, which were not present on the microarray but analyzed separately (Figures 1 and 3), we identified in total 60 immune defense and stress response genes that were differentially regulated in *nub*^*1*^ flies compared with wt. Out of these, 45 (75%) (*P* <0.0002) were up-regulated in carcass samples in *nub*^*1*^ flies, supporting that Nub-PD primarily acts as a repressor of immune- and stress-related genes. A large fraction of the ‘immune defense genes’ have previously been shown to be up-regulated by microbial infections in an NF-κB/Relish dependent manner (Figure 7C). We suggest that Nub-PD plays an important role in repressing immune defense genes, because the presence of commensal microbes will also lead to a low level of constitutive activation of NF-κB/Relish in uninfected conditions. Nub-PD will thereby increase tolerance to the presence of commensal microbes in healthy individuals.

Similar analyses of dissected gut samples also revealed significant enrichment (*P* <0.00001) of genes involved in different types of ‘Response processes’ among the *nub*^*1*^ mis-regulated genes (Additional file 6). Within this combined cluster, consisting of 113 unique genes, 47% were up-regulated in *nub*^*1*^ gut compared to wt (Additional files 6, 7 and 8). Similar to the carcass samples, we identified genes involved in immune recognition, signaling and effector processes, as well as in abiotic stress responses among the *nub*^*1*^ mis-regulated genes (Additional file 7). Combining the sets of over-represented genes in carcass and gut samples involved in immune defense and other response processes, we found an overlap of 140 unique genes that are differentially expressed in *nub*^*1*^ flies compared to wt. A hierarchical cluster analysis of these genes (Additional file 9) shows that immune defense genes cluster together and are over-represented among genes that are up-regulated in *nub*^*1*^ mutants in either carcass or gut, or both.

From this global mRNA analysis, we conclude that numerous genes involved in immune defense reactions are abnormally expressed in *nub*^*1*^ mutant flies. Importantly, essentially all of the differentially regulated immune response genes have previously been experimentally validated for their functional roles in the immune system. In addition to genes clearly involved in the immune defense, the response processes clusters include genes with pertinent roles in cellular stress response processes, such as oxidative stress, hypoxia, heat-shock, DNA damage/repair and apoptosis. This suggests that Nub-PD may not only be involved in protecting flies against aberrant activation of immune system genes but also moderate responses to other types of cellular stress. We believe that Nub-PD acts as a modulator of immune responses, by preventing inappropriate expression of potent effector molecules. The array results also indicate that Nub-PD may play a role in regulation of stress responses, either directly or indirectly, but this assumption needs to be further validated.

### Genes involved in metabolism, development and differentiation require Nub-PD for normal expression

Both in carcass and gut samples, GO processes related to metabolism and catabolism were strongly enriched (in carcass, *P* <0.0001; in gut, *P* <0.00005) among the differentially expressed transcripts in *nub*^*1*^ mutants compared to wt (Additional files 4 and 6). By combining the sets of over-represented genes in carcass and gut samples, we found an overlap of 111 unique genes involved in metabolic and catabolic processes, and differentially expressed by a factor of at least two in *nub*^*1*^ flies compared to its wt counterpart. Of these genes, 48% were down-regulated in gut in *nub*^*1*^ mutants (Additional file 6), indicating an important role for Nub-PD in maintaining normal function in the gut and in metabolic homeostasis.

The role of *nub* in developmental processes during development of larval and adult structures, such as nervous system, wing and leg, is well documented [20,21,23,25-28]. Our gene expression analysis of *nub*^*1*^ mutants revealed that in the adult gut, there is differential expression of similar clusters of genes that previously have been linked to nervous system development (101 genes), development and differentiation of wing and legs (89 genes), and subgroups of these GO terms (Additional file 8). Again, there is much overlap between these groups with 80 unique genes shared between the GO biological processes ‘Development and Differentiation’ and ‘Nervous System’. A large proportion of these genes cluster together (Additional file 10) and are down-regulated in *nub*^*1*^ gut samples, indicating that these genes normally require Nub-PD for their expression in the gut. Of these genes, many are known components of signaling pathways involved in patterning and differentiation of the nervous system in the embryo, and in wing and leg development during metamorphosis. This indicates that the same regulatory systems, involving Nub-PD, also operate in the adult gut, most likely during gut regeneration and differentiation of the gut epithelium.

### Genes that are mis-regulated in *nub*^*1*^ mutants contain Oct sequence motifs

Having demonstrated that Nub protein directly binds to the upstream region of several AMP genes, we decided to perform a survey for Oct sites in the upstream region of genes that were differentially expressed in *nub*^*1*^ compared to wt flies (Figure 7C and Additional file 7). We focused our attention on genes belonging to GO terms related to immune defense, but also included a number of the most differentially expressed non-immune genes, and a few house-keeping genes as controls. In total, 60 genes were searched for the presence of the consensus Oct site AT(C,G)(C,G,T)AAA(A,T) and the Oct-like/nub target motif ATTCAAAT, present in the gene for *Drosophila* choline acetyltransferase [38]. We found a clear correlation between genes that were mis-regulated in *nub*^*1*^ mutants and the presence of Oct sites in the promoter and upstream region (Table 1 and Additional file 11), whereas three typical immune system genes (*Dro6*, *Drs-like* and *Bsk* (*dJNK/MAPK*) whose expressions were not changed in *nub*^*1*^ mutant flies did not contain any Oct sites (Table 1). Four house-keeping or reference genes that did not confer any mis-expression in *nub*^*1*^ mutants were also analyzed, revealing only one Oct site in the distal promoter region of one of these genes. As has been mentioned above, of the genes that showed differential expression in *nub*^*1*^ versus wt flies, and of the genes analyzed here, 71% were up-regulated and out of those, all but two were found to contain at least one consensus Oct site. It is important to note, however, that at least six immune response genes that were down-regulated in *nub*^*1*^ gut tissue also contained Oct sites (Table 1 and Additional file 11), suggesting that Nub-PD regulates constitutive expression of some immune system genes in the gut via Oct sites. In conclusion, *in silico* analysis of gene promoters of genes that are mis-regulated in *nub*^*1*^ flies strongly indicates that Nub-PD directly controls a large group of immune system genes, as well as other genes, via Oct sequence motifs.

**Table 1:** Gene, function and number of Oct and Oct-like/nub sites in the 5′ upstream region

**A. Oct sites in immune and stress response genes with ≥2 fold change in carcass or gut (a)**						
**Gene**	**Computed gene number**	**Function**	**Fold change carcass**	**Fold change gut**	**Oct sites**	**Oct-like /nub**
CecA1	CG1365	Antimicrobial peptide	>10b	>10b	3	2
CecB	CG1878	Antimicrobial peptide	5.5	−1.4	1	
CecC	CG1373	Antimicrobial peptide	13	b.b.	2	
AttC	CG4740	Antimicrobial peptide	45	14	2	1
AttD	CG7629	Antimicrobial peptide	8.1	−1.9		1
Def	CG1385	Antimicrobial peptide	15	1.7	2	
Dpt	CG12763	Antimicrobial peptide	>10b	4b	1	
Dpt B	CG10794	Antimicrobial peptide	2.7	−1.4	3	
Drosocin	CG10816	Antimicrobial peptide	18	4.4	1	1
Drs	CG10810	Antimicrobial peptide	4.9	1.1	1	
Dro2	CG32279	Antimicrobial peptide	b.b	4.1	3	
Dro3	CG32283	Antimicrobial peptide	b.b	2.2	1	
Dro4	CG32282	Antimicrobial peptide	−2.5	−2.2	1	
Dro5	CG10812	Antimicrobial peptide	−1.2	−7.9	2	1
Listericin	CG9080	Antimicrobial peptide	4.4	−1.7	2	
Mtk	CG8175	Antimicrobial peptide	2.5	1.5	3	1
Anp	CG1361	Antimicrobial peptide	b.b	154	5	
LysX	CG9120	Antimicrobial protein	b.b	−17	3	
IM23	CG15066	Immune molecule	21	3.5	2	1
IM10	CG18279	Immune molecule	−20	−26	2	1
PGRP-SC2	CG14745	Recognition	4.8	1.1	3	
PGRP-SB1	CG9681	Recognition	4.8	1.5	1	
PGRP-LD	CG32912-RB/RA/RD	Recognition	4.6	1.8	1	
					2	
PGRP-LC	CG4432	Recognition	3.8	2.9	2	
PGRP-LB	CC14704-RA/RC/RD	Recognition	3.3	2.6	4	
					5	
					4	1
Tep II	CG7052	Recognition	2.7	1.6	2	
Tep IV	CG10363	Recognition	2.4	1.8	4	
GNBP3	CG5008	Recognition	−2.1	−3.9	3	
Sr-CIV	CG3212	Scavenger receptor	b.b	11	1	1
Galpha49B	CG17759-RD/RE	Signaling	2.5	4.2	3	1
Pirk	CG15687	Signaling	2.5	4.3	5	
Prx2540-2	CG11765	Peroxiredoxin	2.0	−1.4	1	
Tsf1	CG6186	Iron sequestration	2.9	2.1	2	
TotX	CG31193	Stress peptide	3.3	2.9	2	
TotM	CG14027	Stress peptide	2.1	2.2	3	
**B. Oct sites in immune genes and in house-keeping genes with no significant fold change in carcass or gut (a)**						
Dro 6	CG32268	Antimicrobial peptide	1.2	1.0	–	–
Drs-like	CG32274	Antimicrobial peptide	1.2	1.1	–	–
Bsk	CG5680	JNK Signaling	1.1	−1.3	–	–
Gapdh1	CG12055	Glycolys	1.2	1.1	–	–
Rpl32/rp49	CG7939	Ribosomal protein	−1.1	−1.1	1	–
Act5C	CG4027	Actin	1.1	1.2	–	–
Aats-arg	CG9020	Arginine tRNA syntase	1.0	−1.1	–	–

Consensus sequence of Oct sites: AT(C,G)(C,G,T)AAA(A,T) and of the Oct-like/nub site: ATTCAAAT. Fold change indicates mRNA expression levels in gut and carcass, comparing wild type versus *nub*^1^ mutant, data taken from the microarray data in Additional files 5 and 8 unless other wise indicated. Non-reliable values due to concentrations below background have been removed. A longer list of analyzed genes including the positions of the Oct sites is presented as Additional file 11. ^a^Location of sites within 2000 bp from the transcription start site of respective gene or up to the nearest exone of an adjacent gene. ^b^Data taken from RT-qPCR results (Figures 1 and 3). b.b., concentrations below background.

## Discussion

Immune defense processes have to be instant and powerful to fight emergent infections efficiently and rescue the host. Minute concentrations of immune elicitors can communicate the presence of foreign organisms, which will lead to coordinated changes in expression of downstream effector genes. However, unprovoked activation of immune responses can be harmful because production of very potent biological effector molecules may cause damage to host tissues. Also, switching of gene regulatory programs may interfere with normal growth, development and other essential processes. Both signaling and gene expression need to be well controlled both before and after the acute stage of an infection, to regulate the rapid and powerful activation followed by attenuation of immune effector gene expression. In *Drosophila*, several negative regulators of immune regulatory pathways have been identified, especially for the IMD pathway [3,4,9]. Very few direct repressors of immune gene transcription have been identified, although it is generally acknowledged that dedicated transcriptional repressors are equally important as upstream regulatory pathways that limit production and activity of transcriptional activators [47]. The homeodomain protein Cad was shown to inhibit Relish-dependent expression of AMP genes in the posterior midgut, and down-regulation of Cad by RNAi promoted over-expression of AMP genes [10]. Similarly, expression of *AttA* was reported to be inhibited by a repressosome complex, consisting of dAP-1, STAT92E and the High Mobility Group (HMG) protein Dsp1 [11]. The present work show that the *Drosophila* POU transcription factor Nub-PD is an important transcriptional repressor of immune defense genes.

Our work suggests that Nub-PD can act both as an activator and a repressor, but the mechanism for this switch is not yet known. It has previously been shown, using transcription assays in *Saccharomyces cerevisiae*, that *Drosophila* Nub-PD binds to Oct sequence motifs to regulate transcription. Direct transactivation capacity was limited, suggesting that co-activators play a role [39]. We show that Nub-PD directly binds to proximal promoter regions of several AMP genes in chromatin prepared from whole flies. These promoter regions contain one or several Oct sites, of which some are overlapping with or located near IRE containing κB and/or GATA sites. It may be possible that the mechanism of Nub-PD repression involves direct competition for binding sequences. However, it may also depend on post-translational modifications, interactions with co-factors and chromatin remodeling complexes.

We show that Nub protein is present in nuclei of fat body cells and in all regions of the gut in healthy flies (Figure 5), which makes it an ideal gate-keeper of immune gene expression. It was previously shown that the presence of peptidoglycan from commensal microbes leads to low levels of constitutive activation of NF-κB/Relish in the midgut [10]. While it can be debated whether the fat body is under continuous immune challenge or not, it has been shown that small peptidoglycan fragments shed from live or dead bacteria can cross barrier epithelia and activate a systemic immune response in the fat body [48-50]. Without protective mechanisms, this would lead to constant activation of immune and inflammatory reactions. Clearly, in the absence of Nub-PD, immune gene expression is over-active both in the gut and in the fat body; remarkably, this leads to serious consequences for the commensal gut flora, which are severely affected in *nub*^*1*^ mutants. We propose that Nub-PD plays an important role in several immunoresponsive tissues by suppressing immune activation and allowing the continuous presence of a commensal gut microbiome. The effects observed in *nub*^*1*^ flies are reminiscent of inflammatory diseases in mammals, in which gut homeostasis is disrupted and the immune system is constantly activated by the presence of the commensal gut flora. The present study indicates that negative regulation by POU/Oct transcription factor(s) are crucial in flies for suppression of immune activation, thereby promoting tolerance to the gut commensal flora. It has not yet been explored if negative regulation by POU/Oct or other transcription factors normally promote tolerance to the commensal flora in healthy mammals, neither has the lack of such negative regulation been surveyed as a possible cause of chronic inflammatory disease establishment.

We show that at least 37 immune defense genes are over-expressed in flies lacking Nub-PD (Table 1 and Additional file 7), suggesting that it serves as a general repressor of immune gene expression in the absence of true infections. Most of the up-regulated genes encode direct effectors of the immune defense, such as AMPs and enzymes that synthesize ROS. This elevated expression of immune defense genes in the gut had a striking effect on the commensal gut flora of *nub*^*1*^ mutants. A majority of the up-regulated genes have previously been shown to be targets of the NF-κB/Relish/IMD pathway or a combination of NF-κB/Relish/IMD and NF-κB/Dif/Toll pathways [44-46]. A few Toll pathway components were found to be down-regulated in *nub*^*1*^ mutants, such as *Persephone*, *GNBP3* and *WntD.* It is important to note that genes encoding negative regulators of the immune response were either up-regulated in *nub*^*1*^ mutants (*PGRP-LB*, *PGRP-SC2*, *PGRP-SB1*, *Pirk*) or not significantly changed (*Dredd*, *Caspar*, *dUSP36*, *DNR1*, *Cad*, *AP1*, *STAT92*), demonstrating that, in the *nub*^*1*^ mutant, the increased expression level of a large number of effectors, such as AMP genes, is not an indirect effect due to decreased expression of the above mentioned negative regulators. We also did not observe any changed expression of co-activators that have been connected with immune pathway signaling or NF-κB-dependent expression (*MED17* (*dTRAP80*), *nejiere* (*dCBP*)*, Akirin* and *Helicase89B*), arguing against indirect effects via these factors. We confirmed that *CecA1* and *Dipt* were also up-regulated in *nub*^*1*^ mutants after treatment with antibiotics (Figure 1G), excluding that excessive expression in *nub*^*1*^ mutants is caused by ongoing infections. The over-expression of these genes was, however, to a large extent NF-κB/Relish-dependent, suggesting that NF-κB/Relish can be activated by the presence of peptidoglycan fragments from dying or dead bacteria. Our conclusion is that over-expression of immune genes in *nub*^*1*^ mutant flies is to a large extent IMD pathway- and NF-κB/Relish-dependent, but did not require presence of live bacteria. Taken together, these results strongly indicate that Nub-PD directly represses a large number of immune system genes in healthy flies.

The whole genome analysis in *nub*^*1*^ mutants revealed a comprehensive picture of the role of Nub-PD in regulation of genes involved in the fly’s immune system. In addition, genes that belong to GO categories of stress response processes and metabolism or catabolism processes were also strongly enriched in *nub*^*1*^ mutants. This is highly reminiscent of results from gene expression profiling carried out with mammalian Oct-1 deficient cells, which showed a clear over-representation of genes involved in cellular, oxidative and metabolic stress responses. The Oct-1 deficient cells were hypersensitive to a number of different stress conditions, indicating that Oct-1 normally controls the activation of such effector genes [51]. In addition, Oct-1 responds to cAMP signaling in pancreatic and intestinal endocrine cells and may play a role in metabolic homeostasis [52]. Interestingly, Oct-1 has been shown to repress cytokine-induced, NF-κB-dependent expression of the genes for E-selectin and vascular cell adhesion molecules [53]. This was shown to be part of a system involving Oct-1 repression of NF-κB target genes involved in inflammatory processes, and to maintain vascular cells in a quiescent state. It is intriguing that both in insects and mammals, repression of NF-κB-dependent target genes by Nub-PD and Oct-1, respectively, seems to be a hallmark of balancing immune, inflammatory and stress responses. We suggest that these evolutionarily related transcription factors are ancient stress sensors that modulate responses and gene activity, and increase the tolerance to both biotic and abiotic stress.

## Conclusions

This work sheds new light on the complex regulation of innate immunity. We show that the POU/Oct transcription factor Nub negatively regulates genes involved in immune responses. Nub-PD protein binds to upstream sequences and represses gene expression of several AMP genes in healthy flies. Importantly, flies that lack expression of Nub-PD protein have a significantly changed gut microbiome, indicating that the maintenance of a normal gut flora is dependent on negative gene regulation by Nub-PD. Whole genome expression data show that a large number of immune- and stress-regulated genes are normally controlled directly or indirectly by Nub-PD, while other groups of genes, involved in development and metabolic processes, require Nub-PD for their normal expression. This demonstrates that Nub, similar to mammalian POU/Oct proteins, is involved in both positive and negative gene regulation. Importantly, our experimental data provide support for the evolutionary conservation of innate immunity between flies and mammals. This conservation involves not only positive regulation by Toll receptor pathways and NF-κB transcription factors upon infection but also includes complex regulation by POU/Oct transcription factors to modulate gene activity in healthy subjects.

## Methods

### Fly stocks, culture and infections

The following fly strains were used: *Oregon*^*R*^ and *w*^*1118*^ were used as wt controls and *nub*^*1*^*b*^*1*^*pr*^*1*^ was used as the *nub*^*1*^ mutant in all experiments*.* Flies for over-expression of Nub-PD (*w*^*1118*^*; UAS-nubRD*) (described below and in Additional file 1) or down-regulation of Nub (*UAS-dsnub*) (VDRC #6218) [54] were crossed with the Gal4 driver lines *c564-Gal4* (*w*^*1118*^*; P{GawB} c564*) (fat body) and *hs-Gal4* (*w;P{w*[*+mc*] *= Gal4-Hsp70.PB}*). Combinations with reporter strains *CecA1-lacZ* pA10 and pA12 [36] and with *Rel*^*E20*^ mutant flies [55] were obtained through conventional crosses. Flies were maintained in mixed female to male populations at 25°C with a 12 h light 12 h dark cycle. Recordings of AMP gene expression were done with exclusively females.

Germ-free flies were established by moving five-day old flies to sterile medium supplemented with a cocktail of antibiotics, and keeping them on this medium for at least seven days, as previously described [10]. The antibiotic cocktail was added during preparation of sterile culture medium to a final concentration of 100 μg/ml carbenicillin, 100 μg/ml neomycin, 50 μg/ml vancomycin, and 100 μg/ml metronidazole. Control flies for this experiment were kept on sterile medium lacking antibiotics.

Microbial infections of flies were done with a mixture of over-night cultures of Gram-positive *Micrococcus luteus* and Gram-negative *Enterobacter cloacae* β12, which were washed once and suspended in PBS (pH7). Five-to-ten day old flies were injected with ≤0.1 μl bacterial suspension per fly using a glass capillary connected to a micro injector (TriTech Research, Los Angeles, CA, USA).

### Plasmids, generation of transgenic flies and cell transfections

Construction of plasmids for the generation of transgenic flies carrying *UAS-nub-RD* was done using the Gateway® Technology (Invitrogen, Carlsbad, CA, USA). Briefly, *nub-RD* cDNA was amplified from the expression plasmid *pAct-Pdm1*[35] using Pfu DNA polymerase (Thermo Fisher Scientific, Waltham, MA, USA) and the following conditions: 95°C for 3 minutes; 95°C for 30 seconds, 60°C for 30 seconds, 72°C for 4 minutes, repeated 30 cycles; 72°C for 5 minutes. The purified PCR product was cloned into the pENTR™/D-TOPO vector using pENTR™ Directional TOPO Cloning (Invitrogen) followed by recombination of the *nub-RD* cDNA into the pTW destination vector, (obtained from TD Murphy), using LR Recombination and the LR Clonase™ enzyme mix (Invitrogen). P-element transformation of *w*^*1118*^ flies was done according to standard protocols [56].

Deletion of the Oct cluster region in the *CecA1* upstream region to create *pA10*△*Oct-luc* was done by inverse PCR with phosphorylated primers using the *pA10-luc* [−751 to +71] construct [7] as starting material. Sequences of primers and details of the PCR protocol are described in the Additional file 12.

Cell transfections were done in *Drosophila* mbn-2 cells at 25°C, using a calcium phosphate transfection kit (Invitrogen), as described previously [8]. The Dual-Luciferase®Reporter Assay System (Promega, Fitchburg, WI, USA) was used to measure the different luciferase values, according to the manufacturer’s instructions. Transfections were done with 1 μg of *pA10-luc* (gift from W-J Lee) or *pA10△Oct-luc* construct (this work) and 100 ng of *PolIII–Renilla luciferase* (Addgene plasmid 37380) [57] (gift from N Perrimon) as internal reference, and mixed with carrier DNA to reach 10 μg.

### Cultivation of bacterial microflora

Flies were anesthetized and sterilized in 70% ethanol for five minutes to eliminate bacteria on the fly surface. Individual guts were dissected under aseptic conditions and homogenized in 100 μl sterile PBS using a plastic pestle. The whole volume of each *nub*^*1*^ gut homogenate was plated out on non-selective lysogeny broth agar plates (1.5% [wt/vol] agar, 1% [wt/vol] tryptone, 0.5% [wt/vol] yeast extract, 1% NaCl), and each *OrR* gut homogenate was diluted 100× in sterile PBS prior to plating. The plates were incubated overnight at 37°C. Because no colonies grew from *nub*^*1*^ homogenates, incubation at room temperature was also tested, which also did not produce any colonies.

### Antibody production, immunostaining and β-galactosidase staining

Antibodies against Nub-PD/PB were raised in rabbits against a synthesized peptide (C-QYKQEEDYDDANGG) (amino acids 119–132) conjugated to keyhole limpet hemocyanine carrier protein (Thermo Fisher Scientific, Waltham, MA, USA). The Nub peptide without the carrier protein was coupled to cyanogen bromide-activated sepharose 4B according the manufacturer’s protocol (Sigma-Aldrich, St Louis, MI, USA), and used for affinity-purification of the antisera.

Antibody staining of dissected larval and adult tissues was performed as described previously [58]. Cryostat sections of adults were prepared essentially as described in [59] with the following adjustments: cryostat sections (20 μM) were cut using a cryomicrotome (Jung CM1800, Leica Microsystems GmbH, Wetzlar, DE). Immunostaining of the sectioned tissues was done as described for dissected tissues. Primary antibody against Nub was used at 4 μg/ml and secondary antibodies were Alexa Fluor 488 conjugated goat anti-rabbit antibody (1:1,000) (Molecular Probes, Eugene, OR, USA). Specimens were analyzed in an Axioplan 2 fluorescence microscope (Carl Zeiss AG, Oberkochen, Germany), documented with a Hamamatsu Orca-ER digital camera (C4742-95) and processed with Axiovision Rel 4.8 software.

For analysis of *CecA1-lacZ* reporter gene expression, flies were dissected, fixed and stained for β-gal activity using 5-bromo-4-chloro-3-indolyl-β-D-galactopyranoside as substrate, as described previously [36].

### Protein extraction and immunoblotting

Protein extraction from the *Drosophila* cell line mbn-2 was done as previously described [60]. Extraction of 30 to 45 dissected intestines were done by homogenizing the tissues with a metal pestle dipped in liquid nitrogen and suspended in a non-denaturing buffer (20 mM 4-(2-hydroxyethyl)-1-piperazineethanesulfonic acid, pH 7.9, 0.56 M potassium chloride, 0.2 mM ethylenediaminetetraacetic acid, 1.5 mM magnesium chloride, 2 mM dithiothreitol, 25% glycerol) supplemented with protease inhibitor according to the manufacturer (Roche Applied Science, Penzberg, Upper Bavaria, Germany). SDS-PAGE was done with 16 to 20 μg of extracted protein per lane. After transfer to polyvinylidinefluoride membranes (Millipore Corporation, Billerica, MA, USA) the membrane was blocked in 10% non-fat dried milk and incubated with anti-Nub antibody (0.1 μg/ml) overnight at 4°C and incubated with SuperSignal West Femto Maximum Sensitivity Substrate (Thermo Scientific) according to the manufacturer’s instruction. Signal quantification was performed using computer software Image J [61].

### RNA extraction and quantitative RT-PCR analysis

RNA extraction, DNase treatment, RT and PCR were carried as previously described [59]. For RT-qPCR analysis, total RNA was isolated from 10 to 30 flies (whole fly extracts) or from 45 dissected guts. When possible, primers and/or probes covered intron/exon boundaries to ensure specific amplification of cDNA; the sequences are given in Additional file 12. All samples were analyzed in triplicate (unless otherwise indicated), and the measured mRNA concentration was normalized relative to the control *RpL32* values. The normalized data were used to quantify the relative levels of a given mRNA according to comparative cycle threshold (2^−ΔΔ^CT) analysis [62,63]. Statistical significance was calculated using paired *t*-test and *P*-values of <0.05, <0.01 and <0.001 were considered significant.

### Chromatin immunoprecipitation

Chromatin was prepared from 20 female flies after manual homogenization and cross-linking with 4% formaldehyde as described previously [64] with the following adjustments: Chromatin fragmentation was done by sonication using a Bioruptur (CosmoBio-Diagenode, Liege, Belgium) for 4 × 10 minutes, to reach DNA size of 100 to 350 bp. Chromatin fragments were incubated with antibodies against Nub (30 μg/ml) or RNA polymerase II C-terminal domain (10 μg/ml) (Ab5408; Abcam, Cambridge, UK) and immune complexes were isolated using 50 μl of a 50% mix of protein A- and protein G-dynabeads (Invitrogen). For immunoprecipitation experiments with naked DNA, phenol-chloroform extraction of proteins was carried out prior to cross-linking and sonication, using standard protocols. The isolated DNA fragments were amplified by PCR using primers specific for *CecA1* (CG1365), *CecC* (CG1373), *AttC* (CG4740), *DiptA* (CG12763) and *Act5C* (CG4027) genes, and for the negative control primers were placed in a non-transcribed intergenic region in chromosome 3L. The PCR conditions were optimized to avoid saturation. Sequences of primers and details of the PCR protocol are described in Additional file 12.

### Microarray analysis, processing and extended analysis

Total RNA was extracted as described [59] from dissected guts and from the rest of the fly minus gut and head (carcass) of 7- to 12-day-old female flies. Isolated RNA was further purified using RNAeasy (Invitrogen) according to manufacturer’s instructions. Tissues from three independent pools of 20 flies were used and analyzed as biological replicates. Pre-processing of the raw-data (Affymetrix .cel files) was done according to the standard analysis pipeline at the Bioinformatics and Expression Analysis Core Facility at Karolinska Institutet, Huddinge, Sweden [65]. Briefly, .cel-files were imported into Affymetrix Expression Console, pre-processed and normalized using the MAS5 default pipeline. No outlier effects were revealed by quality control plots. The data discussed in this publication have been deposited in National Centre for Biotechnology’s Gene Expression Omnibus [66] and are accessible through GEO Series accession number [GSE44234] [67].

After pre-processing and normalization, two-group comparison t-tests were executed on respective sample groups (*nub*^*1*^ and wt, as well as gut and carcass) to identify genes differentially regulated at either the 95% confidence level (*P* <0.05) or the 99% confidence level (*P* <0.01). The raw data were normalized, pre-processed and filtered to remove genes that were not expressed at a detectable level (estimated background signal), leaving 44% and 41% of the transcripts expressed in wt carcass and gut, respectively. For the *nub*^*1*^ sample group, the distribution of expressed probe identities was 45% and 41% for the carcass and gut sample groups, respectively.

A factorial map of principal component analysis was executed on the whole expressed data by the program Qlucore [68] on the differentially expressed (fold change >2). Gene set enrichment analysis to reveal enriched GO biological processes was performed using Cytoscape [69] and the plugin BiNGO [70]. The analysis was executed using the hyper-geometric test with Benjamini-Hochberg false discovery rate correction (*P* <0.05 for gut; *P* <0.01 for gut and carcass).

Hierarchical clustering analyses were performed using Qlucore [68] on the GO hubs ‘Immune System and Response Process’ as well as extracted differentially expressed genes belonging to the GO clusters ‘Development and Differentiation’ and ‘Nervous System’.

For the Venn diagram figures, a web-based program called Venny was used [71].

## Abbreviations

AMP: Antimicrobial peptides; Att: Attacin; B-gal: B-galactosidase; bp: Base pairs; Cad: Caudal; Cec: Cecropin; CFU: Colony-forming Units; ChIP: Chromatin immunoprecipitation; Dif: *Dorsal-*related immunity factor; Dipt: Diptericin; GO: Gene ontology; IMD: Immune Deficiency; IRE: Infection-induced response elements; kDA: kiloDalton; NF: Nuclear factor; PBS: Phosphate-buffered saline; PGRP: Peptidoglycan recognition proteins; RNAi: RNA interference; ROS: Reactive oxygen species; RT-qPCR: quantitative reverse transcription polymerase chain reaction; UTR: Untranslated region; wt: wild type.

## Competing interests

The authors declare that they have no competing interests.

## Authors’ contributions

WD, MMD, XT and YE conceived, designed and performed the experiments. HU, AJ and AB designed and performed initial studies and experiments. JML carried out the extended microarray, statistical and bioinformatic downstream analysis. WD, MMD, JML and YE wrote the paper. All authors read, commented and approved the final manuscript.

## Supplementary Material

Additional file 1 **The *nub *gene organization.** Click here for file

Additional file 2 Nub antibody specificity. Click here for file

Additional file 3 **Expression of *CecA1, Dipt *and *Drs *mRNA is not significantly different in OrR and *nub***^***1 ***^**flies in response to bacterial infection.** Click here for file

Additional file 4 **Gene set enrichment analysis of the 642 differentially expressed transcripts in *nub***^***1 ***^**carcass.** Click here for file

Additional file 5 **Data set of gene set enrichment analysis for *nub***^***1 ***^**versus wild type comparison (carcass).** Click here for file

Additional file 6 **Gene set enrichment analysis of the 961 differentially expressed transcripts in *nub***^***1 ***^**gut.** Click here for file

Additional file 7 **List of ‘Response process’ genes up-or down-regulated in *nub***^***1 ***^**mutants.** Click here for file

Additional file 8 **Gene set enrichment analysis for *nub***^***1 ***^**versus wild type comparison (gut).** Click here for file

Additional file 9 Hierarchical clustering of ‘Immune system and Response processes’ genes. Click here for file

Additional file 10 Hierarchical clustering of ‘Development and Differentiation’ and of ‘Nervous System’ genes. Click here for file

Additional file 11 **Presence of Oct and Oct-like sites in a selection of *nub***^***1***^***- *differentially expressed genes.** Click here for file

Additional file 12 Primer/probes sequences and details of PCR protocols. Click here for file
