# Supplementary material for: The Oct1 homolog Nubbin is a repressor of NF-κB-dependent immune gene expression that increases the tolerance to gut microbiota
Source: BMC Biol. 2013 Sep 6;11:99. doi: 10.1186/1741-7007-11-99 (PMC3849502; doi:10.1186/1741-7007-11-99)
Supplement: Additional file 1 — The nub gene organization. [file 1741-7007-11-99-S1.pdf]

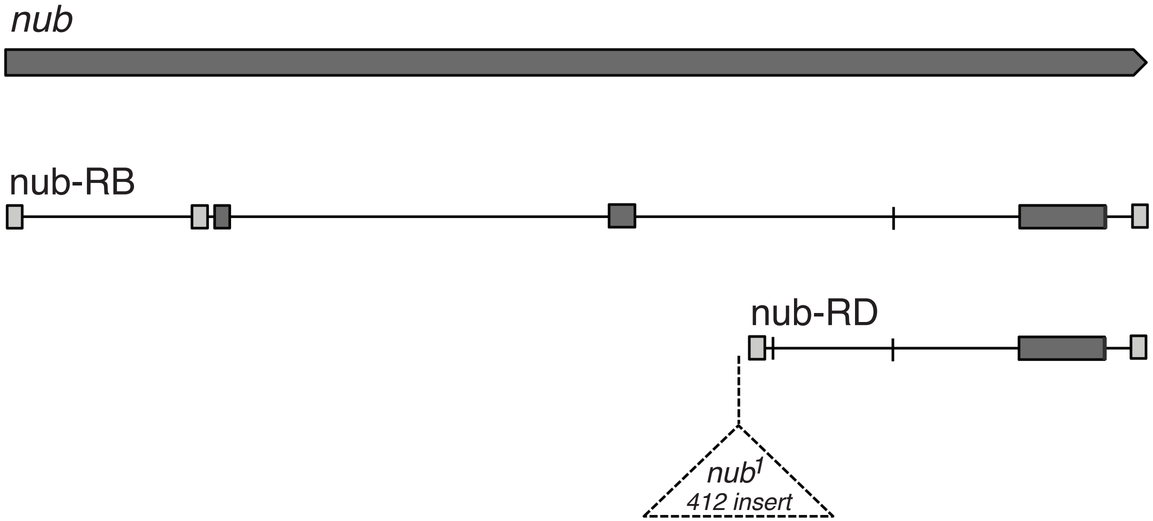

Additional File 1: The *nub* gene organization.
Schematic representation of the nub gene, the two transcription units nub-RB and nub-RD, and the chromosomal rearrangement in *nub1* [20]. The *nub1* mutation is associated with the insertion of the 412 retrotransposon in the second intron of the nub-RB transcription unit, near the first exon of nub-RD. This strongly reduces nub-RD expression in *nub1* mutants [20] and Nub-PD protein is not detected (see Additional File 2).
