# Supplementary material for: The Oct1 homolog Nubbin is a repressor of NF-κB-dependent immune gene expression that increases the tolerance to gut microbiota
Source: BMC Biol. 2013 Sep 6;11:99. doi: 10.1186/1741-7007-11-99 (PMC3849502; doi:10.1186/1741-7007-11-99)
Supplement: Additional file 2 — Nub antibody specificity. [file 1741-7007-11-99-S2.pdf]

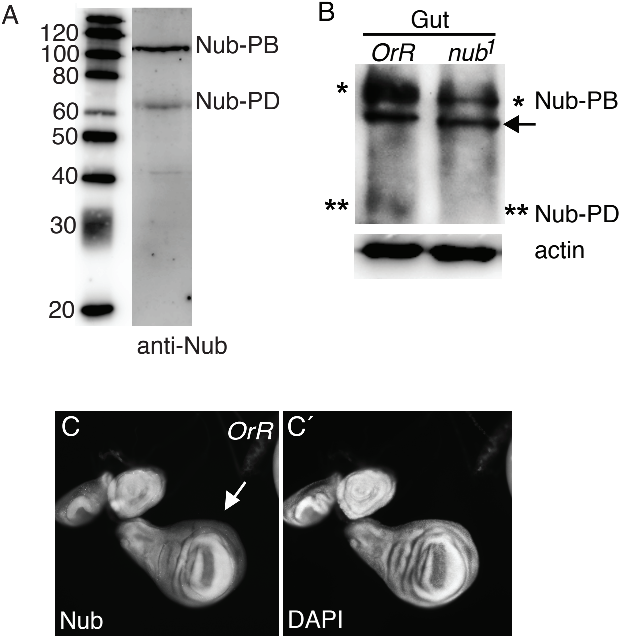

Additional File 2: Nub antibody specificity
(A) Immunoblot experiment confirming the specificity of a new peptide antibody made against the C-terminal part of Nub proteins, and the existence of two predicted protein products of the *nub* gene. Extracts from the *Drosophila* cell line mbn-2 reveal two nub-encoded proteins: Nub-PB (104 kDa) and Nub-PD (65 kDa). **(B)** Immunoblot of gut extracts from wild type *(OrR)* and *nub1* mutants showing the preferential loss of Nub-PD protein (**) in nub1 mutants, while Nub-PB (*) is intact in the mutant. Actin is used as a loading control. The arrow indicates a protein product which does not match the predicted molecular weight of either Nub-PB or Nub-PD
(C) Immunostaining using the peptide-specific Nub antibody confirmed the expected pattern of Nub protein staining in wing (arrow) and leg discs of a wild type larva. Nuclei stained with DAPI (B′).
