# Supplementary material for: The Oct1 homolog Nubbin is a repressor of NF-κB-dependent immune gene expression that increases the tolerance to gut microbiota
Source: BMC Biol. 2013 Sep 6;11:99. doi: 10.1186/1741-7007-11-99 (PMC3849502; doi:10.1186/1741-7007-11-99)
Supplement: Additional file 3 — Expression of CecA1, Dipt and Drs mRNA is not significantly different in OrR and nub1 flies in response to bacterial infection. [file 1741-7007-11-99-S3.pdf]

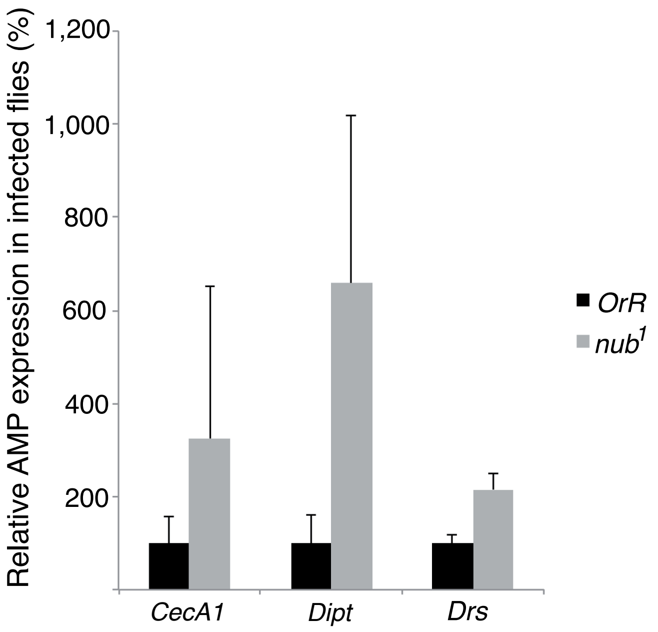

Additional File 3: Expression of *CecA1, Dipt* and *Drs* mRNA is not significantly different in OrR and *nub1* flies in response to bacterial infection.

Quantification by RT-qPCR in extracts of infected whole flies. Expression in *OrR* (set as 100 %) and *nub1* flies three hours post-infection. The data are mean values *(n=3)* and error bars indicate standard deviation. Statistical significance calculated using paired t-test showed that the difference between *OrR* and *nub1* is not significant *(CecA1, p=0.41, Dipt, p= 0.13 and Drs, p=0.055)*.
