# Supplementary material for: The Oct1 homolog Nubbin is a repressor of NF-κB-dependent immune gene expression that increases the tolerance to gut microbiota
Source: BMC Biol. 2013 Sep 6;11:99. doi: 10.1186/1741-7007-11-99 (PMC3849502; doi:10.1186/1741-7007-11-99)
Supplement: Additional file 4 — Gene set enrichment analysis of the 642 differentially expressed transcripts in nub1 carcass. [file 1741-7007-11-99-S4.pdf]

Additional file 4 Gene Set Enrichment Analysis, carcass

	>10		<-10
	5 to 10		-10 to -5
	<5		>-5

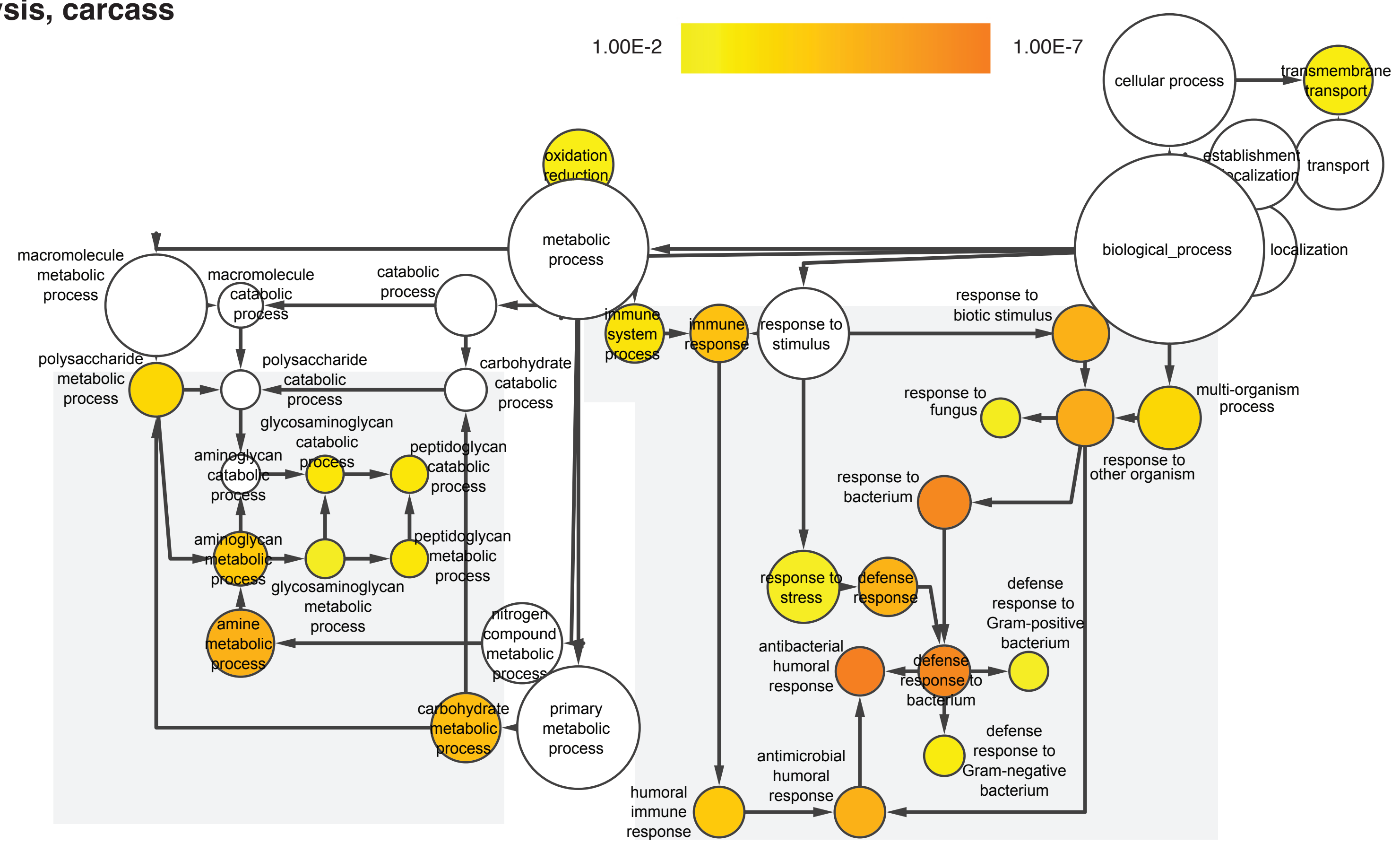

Metabolic and Catabolic processes (51 genes)

LvpH	CG10725	CG18278
CG31148	PGRP-LC	5Ptasel
CG9463	Peritrophin-15a	CG6638
CG14957	CG6733	GNBP3
Uro	Peritrophin-15b	Hex-C
CG11669	Gpdh	CG9232
CG5091	PGRP-LB	CG11357
PGRP-SC2	Muc11A	Mgat2
PGRP-SB1	CG32024	CG9362
CG1544	CG6385	CG5840
PGRP-LD	CG16965	CG7470
Mur18B	CG9510	Pepck
CG17109	CG5493	CG6106
CG13876	CG10154	Tps1
CG7298	CG6465	Odc1
CG8693	rtv	CG4335
PGRP-SD	CG7017	CG14934
		Cht9
		b

Immune system and Response processes (58 genes)

AttC	SPR	Caf1
IM23	GLaz	CG15422
Def	Tsf1	Listericin
CecC	Cyp4d21	CG16756
Dro	Hcs	Fad2
AttD	DptB	GNBP3
lola	TepII	drd
CecB	pirk	tko
gkt	iav	dom
Drs	Mtk	mle
PGRP-SC2	Galpha49B	mthl8
PGRP-SB1	CG32495	dro4
AttA	spirit	Spn
PGRP-LD	phr6-4	phr
Hsp70Ba	TepIV	CG7227
Hsp22	Corp	alph
PGRP-SD	TotM	Hsc70-2
PGRP-LC	IM1	IM10
PGRP-LB	Deaf1	
TotX	Prx2540-2	

Additional file 4: Gene Set Enrichment Analysis (GSEA) of the 642 differentially expressed transcripts in *nub1* carcass.
Differentially expressed transcripts having 2-fold and $p<0.01$ (642) were subjected to GSEA and enriched clusters were extracted. This figure should be statistically interpreted as follows: The nodes, corresponding to different Gene Ontology clusters, are either not colored (white) i.e. not found with any statistical power or colored in the scale yellow to orange, where yellow nodes are found with statistical significance after Bonferroni correction $p<0.01$ and orange colored nodes are found with increasing statistically significance after correction, with a $p<7*10-7$. The size of the circles reflects the number of genes found in each Biological Process, where a smaller circle contains fewer genes than a larger. The grey-zoned data highlights statistically enriched clusters of nodes (genes/transcripts), which all are represented under the manually designated heading e.g. 'Immune system and Response processes' or 'Metabolic and Catabolic processes'. The color-coded table represents the differentially regulated genes found under each grey-zoned heading (different shades of green < -5 fold change, different shades of red > 5 fold change).
