# Supplementary material for: The Oct1 homolog Nubbin is a repressor of NF-κB-dependent immune gene expression that increases the tolerance to gut microbiota
Source: BMC Biol. 2013 Sep 6;11:99. doi: 10.1186/1741-7007-11-99 (PMC3849502; doi:10.1186/1741-7007-11-99)
Supplement: Additional file 6 — Gene set enrichment analysis of the 961 differentially expressed transcripts in nub1 gut. [file 1741-7007-11-99-S6.pdf]

Additional File 6

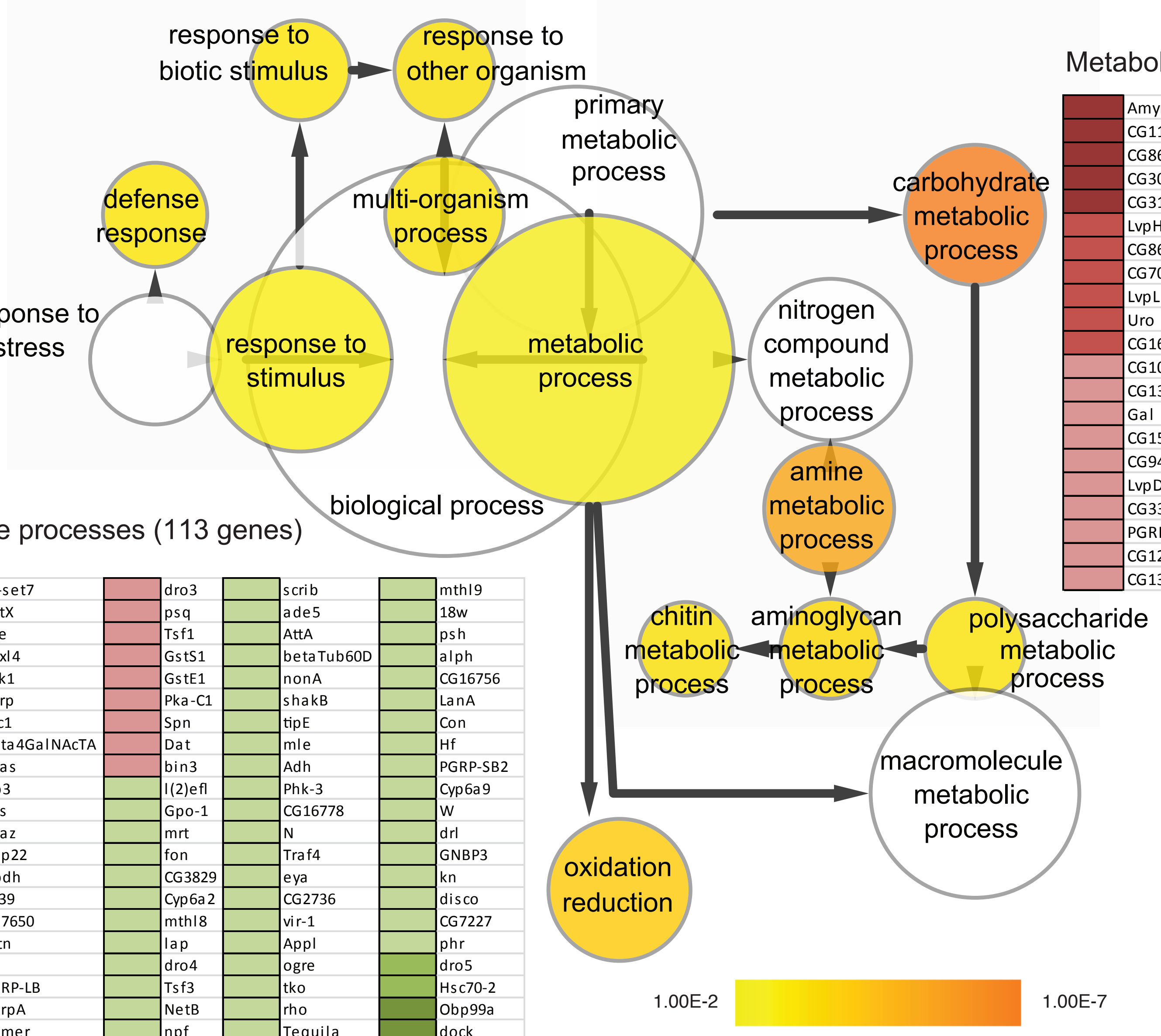

Metabolic and Catabolic processes (81 genes)

Amyrel	CG9674	CG11357	Nmdmc
CG11669	Ace	Hn	Tequila
CG8693	Ssadh	Eip71CD	Idgf5
CG30360	FucTC	Gpo-1	CG5171
CG31148	CG10467	CG14935	PGRP-SB2
LvpH	CG10725	CG10924	GNBP3
CG8690	beta4GalNAcTA	CG14681	Gasp
CG7017	CG4835	Ect3	Odc1
LvpL	CG9547	SamDC	v
Uro	CG11796	fbp	CG4335
CG16965	botv	CG5731	kkv
CG10154	Gpdh	CG31016	CG3348
CG13876	Inos	CG3999	CG13676
Gal	PGRP-LB	CG6188	pip
CG15093	CG6933	obst-B	obst-A
CG9463	Mur18B	CG9362	Cda4
LvpD	ry	CG6415	Cht7
CG33090	CG1544	CG34452	Cht9
PGRP-LC	alpha-Man-I	Faa	
CG1271	Dat	arg	
CG13075	CG7298	Cda5	

>10	<-10
5 to 10	-10 to -5
<5	>-5

Response processes (113 genes)

Anp	pr-set7	dro3	scrib	mthl9
sgg	TotX	psq	ade5	18w
AttC	Ace	Tsf1	AttA	psh
Sr-CIV	Fbxl4	GstS1	betaTub60D	alph
scb	Ork1	GstE1	nonA	CG16756
SPR	Corp	Pka-C1	shakB	LanA
CG32495	Arc1	Spn	tipE	Con
Cyp4d21	beta4GalNAcTA	Dat	mle	Hf
lola	spas	bin3	Adh	PGRP-SB2
lcs	Ilp3	l(2)efl	Phk-3	Cyp6a9
pirk	Hcs	Gpo-1	CG16778	W
Galpha49B	NLaz	mrt	N	drl
dro2	Hsp22	fon	Traf4	GNBP3
gkt	Gpdh	CG3829	eya	kn
Cyp4ac2	Hr39	Cyp6a2	CG2736	disco
Cyp6a8	CG7650	mthl8	vir-1	CG7227
shn	retn	lap	Appl	phr
Est-6	Bx	dro4	ogre	dro5
IM23	PGRP-LB	Tsf3	tko	Hsc70-2
Caf1	norpA	NetB	rho	Obp99a
Lectin-galC1	homer	npf	Tequila	dock
PGRP-LC	TotM	emc	Ilp6	lectin-28C
				LysX
				Cdk5alpha
				IM10

Additional file 6. GSEA of the 961 differentially expressed transcripts in *nub1* gut. Differentially expressed transcripts (961) were subjected to GSEA and enriched clusters were extracted. This figure should be statistically interpreted as follows: The nodes, corresponding to different Gene Ontology clusters, are either not colored (white) i.e. not found with any statistical power or colored in the scale yellow to orange, where yellow nodes are found with statistical significance after Bonferroni correction $p<0.01$ and orange colored nodes are found with increasing statistically significance after correction, with a $p<7*10-7$. The size of the circles reflects the number of genes found in each Biological Process, where a smaller circle contains less genes than a larger. The grey-zoned data highlights statistically enriched clusters of nodes (genes/transcripts), which all are represented under the manually designated heading e.g. 'Response processes' or 'Metabolic and Catabolic processes'. The color-coded table represents the differentially regulated genes found under each grey-zoned heading (different shades of green < -5 fold change, different shades of red > 5 fold change). The three GO processes, including multi-organism process (38 genes), response to biotic stimulus (24 genes) and defense response genes (27 genes) together with additional nodes with similar functions and genes, resulted in a list of 113 unique genes all of which we here call 'Response processes' and this combined cluster is significantly enriched ($p<0.00001$).
