# Supplementary material for: The Oct1 homolog Nubbin is a repressor of NF-κB-dependent immune gene expression that increases the tolerance to gut microbiota
Source: BMC Biol. 2013 Sep 6;11:99. doi: 10.1186/1741-7007-11-99 (PMC3849502; doi:10.1186/1741-7007-11-99)
Supplement: Additional file 7 — List of ‘Response process’ genes up-or down-regulated in nub1 mutants. [file 1741-7007-11-99-S7.pdf]

Additional File 7. List of 'Response process' genes up-or down-regulated in *nub1* mutants.

Gene symbol	Fold change	Function	NF-κB regulated
Anp	154,14	Antimicrobial peptide	
sgg	18,36	Protein kinase, signaling	
AttC	13,66	Antimicrobial peptide	YES
Sr-CIV	11,17	Scavenger receptor	
scb	7,49	Integrin binding, stress response	
SPR	7,27	Sex peptide receptor	
CG32495	7,27	Glutathione synthase	
lola	5,94	Protein binding	
sesB	5,38	Stress response	
pirk	4,30	IMD pathway signaling	YES
Galpha49B	4,17	Gaq, ROS production	
dro2	4,11	Antimicrobial peptide	
gkt	3,90	Nuclease, DNA damage response	
Cyp4ac2	3,78	Cytochrome P450	
Cyp6a8	3,71	Cytochrome P450	
shn	3,58	Transcriptional co-activator	
Est-6	3,54	Carboxylesterase	
IM23	3,52	Immune Molecule	YES
Caf1	3,46	Stress response	
Lectin-galC1	2,95	C-type lectin, recognition	
PGRP-LC	2,93	Immune recognition	YES
pr-set7	2,92	Histone methyltransferase	
TotX	2,89	Stress peptide	
Ace	2,76	Acetylcholinesterase	
Fbxl4	2,74	Leucine-rich repeat protein	
Ork1	2,73	Potassium ion leak channel activity	
Corp	2,57	Apoptosis, DNA damage response	
Arc1	2,56	Nucleic acid binding	
beta4GalNAcTA	2,52	Galactosyltransferase	
spas	2,45	ATPase	
Ilp3	2,45	insulin-like peptide	
Hcs	2,44	Biotin ligase, heat response	
NLaz	2,39	Lipid binding, Stress response	
Hsp22	2,38	Heat shock protein	
Gpdh	2,28	Glycerol-3-phosphate dehydrogenase	
Hr39	2,28	Transcription factor	
CG7650	2,28	Thioredoxin-like protein	

Gene symbol	Fold change	Function	NF-κB regulated
retn	2,27	Transcription factor	
Bx	2,27	Zink finger protein	
PGRP-LB	2,26	Immune recognition	YES
norpA	2,26	Phospholipase C	
homer	2,25	Protein binding, ethanol response	
TotM	2,23	Stress peptide	YES
dro3	2,19	Antimicrobial peptide	
psq	2,18	DNA binding, chromatin silencing	
Tsf1	2,09	Iron sequestration	YES
GstS1	2,09	Glutathione S-transferase	
GstE1	2,09	Glutathione S-transferase	
Pka-C1	2,07	cAMP-dependent protein kinase	
Spn	2,05	Protein phosphatase binding	
Dat	2,04	Acyl-CoA N-acyltransferase	
bin3	2,00	RNA binding	
l(2)efl	-2,06	HSP20-like chaperone	
Gpo-1	-2,08	Glycerophosphate oxidase-1	
mrt	-2,09	Unknown	
fon	-2,09	Hemolymph coagulation	
CG3829	-2,12	Scavenger receptor activity	
Cyp6a2	-2,16	Cytochrome P450	
mthl8	-2,19	G protein coupled receptor activity	
lap	-2,19	Clathrin binding	
dro4	-2,20	Antimicrobial peptide	
Tsf3	-2,21	Iron sequestration	
NetB	-2,23	Axon guidance	
npf	-2,26	Neuropeptide F	
emc	-2,30	Protein binding transcription factor	
scrib	-2,30	Protein binding	
ade5	-2,30	AIR-carboxylase-SAICAR synthetase	
AttA /// AttB	-2,35	Antimicrobial peptide	YES
betaTub60D	-2,36	Beta tubulin	
nonA	-2,37	mRNA binding	
shakB	-2,39	Gap junction channel activity	
tipE	-2,40	Sodium channel regulator activity	
mle	-2,45	Chromatin binding	
Adh /// Adhr	-2,48	Alcohol dehydrogenase	
Phk-3	-2,49	ser/thr kinase activity	

Gene symbol	Fold change	Function	NF-κB regulated
CG16778	-2,51	DNA binding, BTB/POZ-domain	
N	-2,51	Notch, protein binding	
Traf4	-2,54	TNF-receptor-associated factor 4	
eya	-2,59	Protein tyrosine phosphatase activity	
CG2736	-2,65	Scavenger receptor activity	
vir-1	-2,69	Virus-induced RNA 1	
Appl	-2,72	Beta-amyloid precursor protein	
ogre	-2,76	Gap junction channel activity	
tko	-2,94	Ribosomal protein S12	
rho	-3,02	Rhomboid, EGF-R	YES
Tequila	-3,03	Endopeptidase activity, chitin binding	
Ilp6	-3,08	Insulin-like peptide	
mthl9	-3,10	G-protein coupled receptor activity	
18w	-3,10	Transmembrane receptor, TLR	
psh	-3,30	peptidase, immune response	
alph	-3,40	Negative regulator of RAS signaling	
CG16756	-3,44	Lysozyme-like	YES
LanA	-3,54	Laminin A, receptor binding	
Con	-3,54	Homophilic cell adhesion	
Hf	-3,58	Cytokine-like	
PGRP-SB2	-3,67	Immune recognition	
Cyp6a9	-3,70	Cytochrome P450	
W	-3,73	Apoptosis regulation	
drl	-3,79	Protein kinase, Wnt protein binding	
GNBP3	-3,89	Immune recognition	
kn	-4,00	Transcription factor	
disco	-4,08	Transcription factor	
CG7227	-4,21	Scavenger receptor activity	
phr	-4,91	Photolyase, DNA damage repair	
dro5	-7,89	Antimicrobial peptide	
Hsc70-2	-8,31	Heat shock protein family	YES
Obp99a	-10,60	Odorant binding protein	
dock	-12,21	Insulin receptor binding	
ninaE	-16,55	G protein-coupled receptor	
LysX	-16,87	Lysozyme	
Cdk5alpha	-20,53	Cyclin-dependent kinase 5 activator	
IM10	-26,33	Immune Molecule	YES

Names and functions of the 113 genes constituting the enriched Biological Process cluster 'Response processes' identified by the GSEA (Add. File 6) in gut from *nub1* mutants. Fold change (mean values) of mRNA levels is indicated by numbers and color-coded (up-regulation/red; down-regulation/green). Genes that previously have been shown to be targets of the NF-κB/Relish/IMD pathway or a combination of NF-κB/Relish/IMD and NF-κB/Dif/Toll pathways [53-55] are indicated by 'YES'.
