# Supplementary figures and images for: The Oct1 homolog Nubbin is a repressor of NF-κB-dependent immune gene expression that increases the tolerance to gut microbiota
Source: BMC Biol. 2013 Sep 6;11:99. doi: 10.1186/1741-7007-11-99 (PMC3849502; doi:10.1186/1741-7007-11-99)

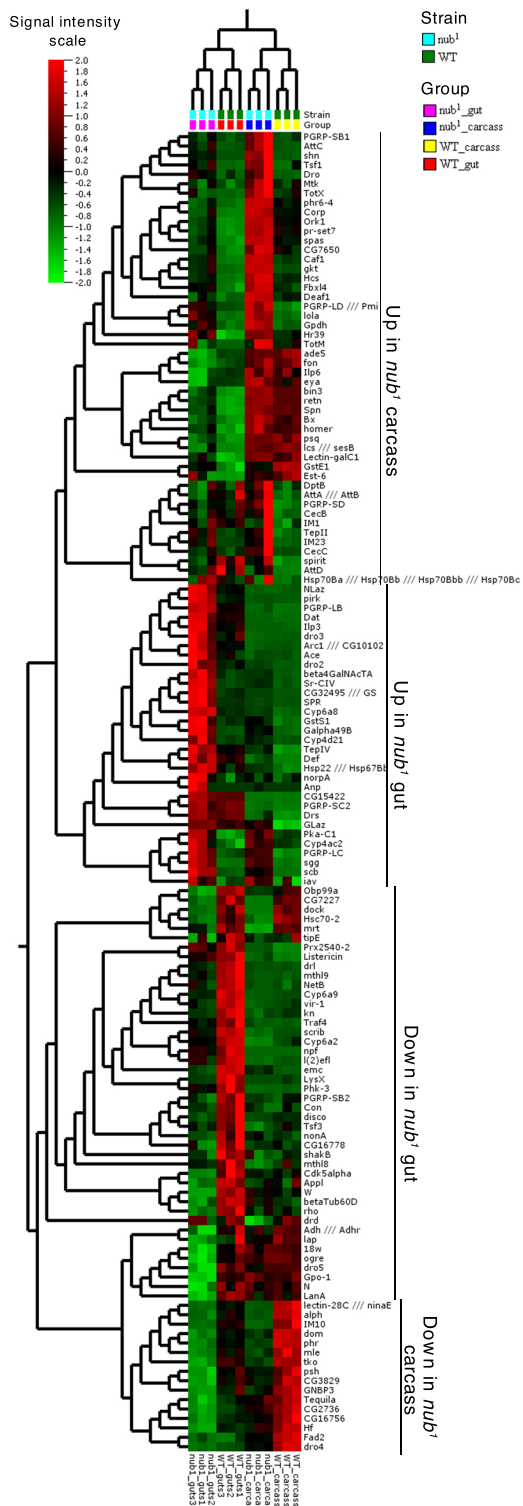

Supplement: Additional file 9 — Hierarchical clustering of ‘Immune system and Response processes’ genes. [file 1741-7007-11-99-S9.pdf]
