# Supplementary material for: The Oct1 homolog Nubbin is a repressor of NF-κB-dependent immune gene expression that increases the tolerance to gut microbiota
Source: BMC Biol. 2013 Sep 6;11:99. doi: 10.1186/1741-7007-11-99 (PMC3849502; doi:10.1186/1741-7007-11-99)
Supplement: Additional file 10 — Hierarchical clustering of ‘Development and Differentiation’ and of ‘Nervous System’ genes. [file 1741-7007-11-99-S10.pdf]

Additional file 10A

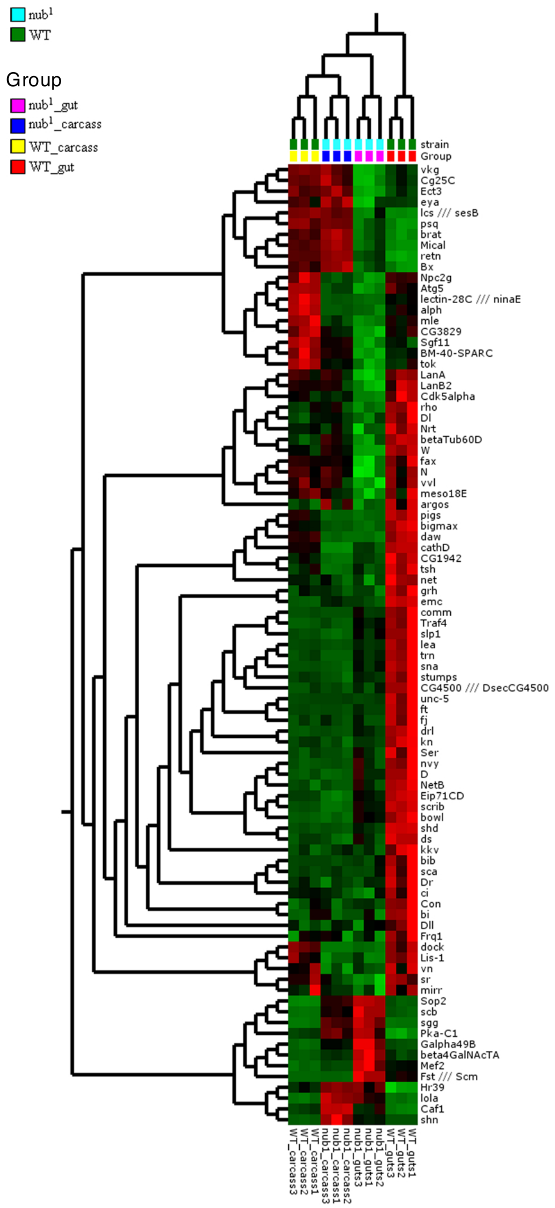

Signal intensity scale

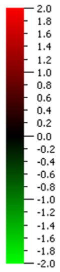

Additional file 10B

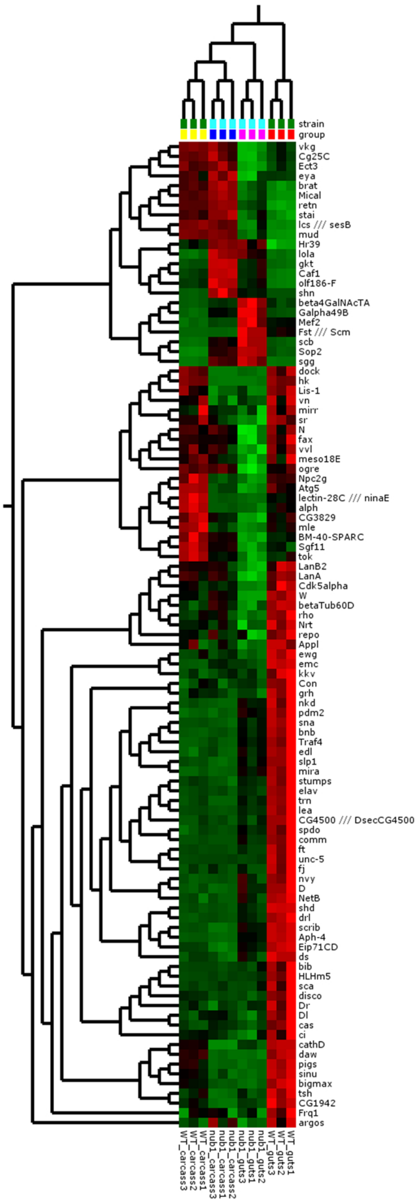

Additional file 10. Hierarchical clustering of 'Development and Differentiation' and of 'Nervous system' genes.

Genes having a similar signal expression pattern are found to cluster together. The signal intensities ranges from red to green where red corresponds to the highest signal expression and green to the lowest.

(A) 'Development and Differentiation': Here we have grouped the following GO Biological processes into a larger group 'Development and Differentiation', all of which have an extensive gene overlap between them. Appendage segmentation, 'leg segmentation', 'leg morphogenesis', 'limb morphogenesis', 'limb development', 'leg joint morphology', 'imaginal disc patterning' and 'appendage development'. **(B)** 'Nervous system': The following GO groups fall within the 'Nervous system' description: 'Axon Guidance', 'Axonogenesis', 'Ganglion mother cell development' and 'Nervous system development'.

** Note: 'cell morphogenesis involved in differentiation' and 'tissue development' are two groups that we believe both fall within the descriptions 'Development and Differentiation' (A) as well as 'Nervous system' (B) and therefore these genes were listed in both of the figures.*
