# Supplementary material for: The Oct1 homolog Nubbin is a repressor of NF-κB-dependent immune gene expression that increases the tolerance to gut microbiota
Source: BMC Biol. 2013 Sep 6;11:99. doi: 10.1186/1741-7007-11-99 (PMC3849502; doi:10.1186/1741-7007-11-99)
Supplement: Additional file 11 — Presence of Oct and Oct-like sites in a selection of nub1- differentially expressed genes. [file 1741-7007-11-99-S11.pdf]

Additional file 11. Presence of Oct and Oct-like sites in a selection of *nub1*- differentially expressed genes.

Immune system genes with >2 fold change in carcass or gut, or both							
Gene	CG number	Fold change carcass (b)	Fold change gut (b)	Oct sites	Position (a)	Oct-like /nub	Position (a)
AttC	CG4740	45	14	2	–861<br>–22	1	–372
IM23	CG15066	21	3.5	2	–237<br>–220	1	–231
Def	CG1385	15	1.7	2	–492<br>–271		
Drosocin	CG10816	18	4.4	1	–315	1	–315
CecC	CG1373	13	(2.0)	2	–644<br>–239		
AttD	CG7629	8.1	–1.9			1	–394
Dro2	CG32279	(8.1)	4.1	3	–676<br>–506<br>–290		
Iola	CG12052	8.0	5.9	n.d		n.d.	
Sr-CIV	CG3212	(6.0)	11	1	–513	1	–870
CecB	CG1878	5.5	–1.4	1	–526		
Drs	CG10810	4.9	1.1	1	–167		
PGRP-SC2	CG14745	4.8	1.1	3	–1891<br>–1175<br>–1063		
PGRP-SB1	CG9681	4.8	1.5	1	–355		
AttA/AttB	CG10146/ CG18372	4.7	–2.5	1	–282		
PGRP-LD	CG32912-RB/RA/RD	4.6	1.8	1<br>2	5' RB –307<br>5' RD –431<br>5' RD –46		
Listericin	CG9080	4.4	–1.7	2	–1438<br>–663		
PGRP-SD	CG7496	3.9	–1.3	–			
PGRP-LC	CG4432	3.8	2.9	2	–1281<br>–213		
TotX	CG31193	3.3	2.9	2	–336<br>–324		
PGRP-LB	CC14704-RA/RC/RD	3.3	2.6	4<br>5<br>4	5' RA –1939<br>5' RA–1226<br>5' RA –902<br>5' RA –149<br>5' RC–1889<br>5' RC –1799<br>5' RC –1730<br>5' RC –1102<br>5' RC –613<br>5' RD –1628<br>5' RD –1265<br>5' RD –1215<br>5' RD –440	1	–1719
Tsf1	CG6186	2.9	2.1	2	–1118<br>–490		
Dpt B	CG10794	2.7	–1.4	3	–849<br>–398<br>–219		
Tep II	CG7052	2.7	1.6	2	–777<br>–250		
Pirk	CG15687	2.5	4.3	5	–1954<br>–1764<br>–1561<br>–809<br>–560		
Galpha49B-5' of RD/RE	CG17759	2.5	4.2	3	–1326<br>–1326<br>–1200	1	–1273
Mtk	CG8175	2.5	1.5	3	–1348<br>–789<br>–212	1	–1240
Tep IV	CG10363	2.4	1.8	4	–1304<br>–1068<br>–24<br>14		
Spirit	CG2056	2.4	–1.8	–		–	
Anp	CG1361	(14.5)	154	5	–448<br>–430<br>–278<br>–116<br>–92		
TotM	CG14027	2.1	2.2	3	–938<br>–782<br>–118		
Prx2540-2	CG11765	2.0	–1.4	1	–1028		
Dro3	CG32283	(–1.7)	2.2	1	–235		

Immune system genes not on the microarray (RT-qPCR)							
Gene	CG number	Fold change carcass (b)	Fold change gut (b)	Oct sites	Position (a)	Oct-like /nub	Position (a)
CecA1	CG1365	>10*	>10*	3	–336<br>–181<br>–174	2	–303<br>–226
CecA2	CG1367	n.d.	n.d	1	–149	2	–324<br>–227
Dpt (c)	CG12763	>10*	4*	1	–153		

Immune system genes with <-2 fold change in carcass or gut, or both							
Gene	CG number	Fold change carcass (b)	Fold change gut (b)	Oct sites	Position (a)	Oct-like /nub	Position (a)
GNBP3	CG5008	–2.1	–3.9	3	–1602<br>–1060<br>–706		
Dro4	CG32282	–2.5	–2.2	1	–93		
Dro5	CG10812	–1.2	–7.9	2	–481<br>–299	1	–460
CG7227	CG7227	–3.8	–4.2	2*	2728<br>1221		
LysX	CG9120	(–18)	–17	3	–526<br>–387<br>–373		
IM10	CG18279	–20	–26	2	–1939<br>–1579	1	–1009

Immune system genes with no fold change on the microarray							
Gene	CG number	Fold change carcass (b)	Fold change gut (b)	Oct sites	Position (a)	Oct-like /nub	Position (a)
Dro 6	CG32268	1.2	1.0	–		–	
Drs-like	CG32274	1.2	1.1	–		–	
Bsk	CG5680	1.1	–1.3	–		–	

Other types of genes with high fold change on the microarray							
Gene	CG number	Fold change carcass (b)	Fold change gut (b)	Oct sites	Position (a)	Oct-like /nub	Position (a)
Cyp12d1-d	CG30489	45	6.7	5	–1280<br>–1111<br>–879<br>–374<br>–125		
CG42335/ CG13420	CG42335/ CG13420	20	11	1	–477		
Ugt86Dd	CG6633	20	8.5	2	–511<br>–151		
CG1304	CG1304	(1.2)	296	3	–1854<br>–713<br>–354	1	–1972
Lip3	CG8823	(3.6)	65	2	–201<br>–146		
CG13335	CG13335	–4.2	–125	2	–746<br>–734		
Cht9	CG10531	–6.2	–67	3	–1035<br>–594<br>–301		
CG11893	CG11893	–8.2	–93	2	–461<br>–98		
CG31775	CG31775	–17	–44	4	–1829<br>–1761<br>–1296<br>54		
Dock	CG3727	–26	–12	3	17<br>2002<br>3198		
Stet/Rho-2	CG33166-RA+ RB	–42	–8.6	3+3	RA –1858<br>RA –1648<br>RA –14<br>RB –1336<br>RB –1215<br>RB –756		
Cyp4p1	CG10842	–51	–9.5	2	–1964<br>–1899	1	–208

House-keeping genes / reference genes							
Gene	CG number	Fold change carcass (b)	Fold change gut (b)	Oct sites	Position	Oct-like /nub	Position (a)
Gapdh1	CG12055	1.2	1.1	–			
Rpl32/rp49	CG7939	–1.1	–1.1	1	–702		
Act5C	CG4027	1.1	1.2	–			
Aats-arg	CG9020	1.0	–1.1	–			

Gene name, CG number, number and position of Oct and Oct-like/nub sites in the 5´upstream region within 2000 bp from the transcription start site of respective gene or up to the nearest exon of an adjacent gene. Consensus sequence of Oct sites: AT(C,G)(C,G,T)AAA(A,T) and of the Oct-like/nub site: ATTCAAAT. Genes that were not analyzed, due to complicated gene structure with multiple transcription start sites, are indicated as not determined (n.d.).

(a) Numbers refer to distance in base pairs relative to the transcription initiation site/cap site.
(b) Fold change indicates mRNA expression levels in gut and carcass, comparing wild type versus nub1 mutant, data taken from the microarray data (Additional files 5 and 8) or from RT-qPCR results (asterisk) (Figs 1 and 3). Numbers in parenthesis indicate non-reliable values (concentrations below background level).
(c) ProbeSet on Affy2.0 microarrays for Dipt: 1 out of 14 probes partially aligns to an exonic region of transcript isoform of Dipt meaning that Dipt cannot be measured reliably on the microarray. Numbers given here are from data in Fig 1 and Fig 3.
