# Supplementary material for: The Oct1 homolog Nubbin is a repressor of NF-κB-dependent immune gene expression that increases the tolerance to gut microbiota
Source: BMC Biol. 2013 Sep 6;11:99. doi: 10.1186/1741-7007-11-99 (PMC3849502; doi:10.1186/1741-7007-11-99)
Supplement: Additional file 12 — Primer/probes sequences and details of PCR protocols. [file 1741-7007-11-99-S12.pdf]

Additional File 12. Primer/probes sequences and details of PCR protocols

Sequences of probes/primers for RT-qPCR:

AttC (CG4740): Dm01821390_g1 (Applied Biosytems)

CecA1 (CG1365): 6-FAM-CAACGCGTTCGATTTT-MGB, F 5′-CTGGGTGGCTGAAGAAAATTGG-3′, R 5′- ATTGTGGCATCCCGAGTGT-3′

CecC (CG1373): Dm02151846_gH (Applied Biosystems)

DptA (CG12763): 6-FAM-CCGATGCCCGACGACAT-MGB, F 5′-GCAATCGCTTCTACTTTGGCTTAT-3′, R 5′-GTGGAGTGGGCTTCATGGT-3′

Drs (CG10810): 6-FAM-CCAGGACCACCAGCATC-MGB, F 5′-GTGAGAACCTTTTCCAATATGATGCA-3′, R 5′-CGGCATCGGCCTCGTT-3′

Def (CG1385): Dm01818074_s1 (Applied Biosystems)

nub-RD (CG34395-PD): Dm01841366_m1 (Applied Biosystems)

RpL32 (CG7939): 6-FAM-CATTTGTGCGACAGCTT-MGB, F 5′-CACCAGTCGGATCGATATGCT-3′, R 5′-ACGCACTCTGTTGTCGATACC-3′

Chromatin immunoprecipitation/Sequences of primers and details of the PCR protocol:

The immunoprecipitated DNA was analyzed by semiquantitative PCR using 2.2 ng DNA template, specific primers, and 5 PRIME HotMasterMix (5 Prime) following the manufacturers instructions. The PCR products were resolved in agarose gels, stained with ethidium bromide, and analyzed using Quantity One v. 4.5.2 (Bio Rad, Hercules, CA, USA). The following primers were used to amplify fragments:

CecA1: F 5′-GTAGTGATTCCCCTCGAAAA-3′

CecA1: R 5′-GACAGATAAGGCATGCACGG-3′

CecC: F 5′-CATCCCCCTCTCAAAAATCA-3′

CecC R 5′-GTAAAAATGGAGCGTTCTTCGCTTA-3′

DptA: F 5′-CCTTTGCTGCAGTTTCGATG-3′

DptA: R 5′-GTTTAAGAAAGATCCCCTGGTGGT-3′

AttC: F 5′-CTATTAAAATTTGAACTTACTCATTGGC-3′

AttC: R 5′-GAGAGATTGTGCATCCCCTTGA-3′

Act5C intron 2: F 5′-CATTTTAAGGGCGCATACACACA-3′

Act5C intron 2: R 5′-CAATACGAATATGCCGAAAAGGG-3′

Intergenic region: F 5′-ATTACAACACCAATGTTAGGTTTAACG-3′,

Intergenic region: R 5′-GAATTGCTTGTTGCGGCTA-3′.

Plasmid construction, sequences of primers and details of the PCR protocol

The Oct deletion construct pA10ΔOct-luc was created by inverse PCR using unphosphorylated primers CecA1ΔOct-F 5′ (TCCCTAGATGTGCAGATGTGTG) and CecA1ΔOct-R 5′ (TTTACATAAACGCATCCAATTTG). The PCR consisted of the following: 98°C for 30 s, followed by 33 cycles of 98 °C for 30 s, 66 °C for 30 s, 72 °C for 2 min, and a final extension of 7 min at 72 °C, using Phusion high-fidelity DNA polymerase (Finnzymes). PCR products were gel purified and ligated overnight at 16 °C, then transformed into chemically competent DH5α cells (Invitrogen), and isolated plasmids were sequenced to confirm recovery of the desired deletion.
